# The non-coding snRNA *7SK* controls transcriptional termination, poising, and bidirectionality in embryonic stem cells

**DOI:** 10.1186/gb-2013-14-9-r98

**Published:** 2013-09-17

**Authors:** Gonçalo Castelo-Branco, Paulo P Amaral, Pär G Engström, Samuel C Robson, Sueli C Marques, Paul Bertone, Tony Kouzarides

**Affiliations:** 1 The Gurdon Institute, University of Cambridge, Tennis Court Road, Cambridge CB2 1QN, UK; 2 European Molecular Biology Laboratory, European Bioinformatics Institute, Wellcome Trust Genome Campus, Cambridge CB10 1SD, UK; 3 Genome Biology and Developmental Biology Units, European Molecular Biology Laboratory, Meyerhofstraße 1, 69117 Heidelberg, Germany; 4 Wellcome Trust – Medical Research Council Cambridge Stem Cell Institute, University of Cambridge, Tennis Court Road, Cambridge CB2 1QN, UK; 5 Laboratory of Molecular Neurobiology, Department of Medical Biochemistry and Biophysics, Karolinska Institutet,SE-17177 Stockholm, Sweden; 6 Present address: Department of Biochemistry and Biophysics, Science for Life Laboratory, Stockholm University, Box 1031, SE-17121 Solna, Sweden

## Abstract

**Background:**

Pluripotency is characterized by a unique transcriptional state, in which lineage-specification genes are poised for transcription upon exposure to appropriate stimuli, via a bivalency mechanism involving the simultaneous presence of activating and repressive methylation marks at promoter-associated histones. Recent evidence suggests that other mechanisms, such as RNA polymerase II pausing, might be operational in this process, but their regulation remains poorly understood.

**Results:**

Here we identify the non-coding snRNA *7SK* as a multifaceted regulator of transcription in embryonic stem cells. We find that *7SK* represses a specific cohort of transcriptionally poised genes with bivalent or activating chromatin marks in these cells, suggesting a novel poising mechanism independent of Polycomb activity. Genome-wide analysis shows that *7SK* also prevents transcription downstream of polyadenylation sites at several active genes, indicating that *7SK* is required for normal transcriptional termination or control of 3′-UTR length. In addition, *7SK* suppresses divergent upstream antisense transcription at more than 2,600 loci, including many that encode divergent long non-coding RNAs, a finding that implicates the *7SK* snRNA in the control of transcriptional bidirectionality.

**Conclusions:**

Our study indicates that a single non-coding RNA, the snRNA *7SK*, is a gatekeeper of transcriptional termination and bidirectional transcription in embryonic stem cells and mediates transcriptional poising through a mechanism independent of chromatin bivalency.

## Background

Pluripotent cells such as embryonic stem cells (ESCs) are able to generate all the cell types of the adult organism, and thus can acquire different cell fates upon appropriate stimuli. The majority (85%) of annotated genes in ESCs experience transcription by RNA polymerase II (Pol II) [1]. Nevertheless, only a subset of these genes is expressed in a robust manner, and Pol II has been reported as being paused at 39% of the annotated genes [1]. Transcription start sites (TSSs) of many genes that are expressed at very low levels are bivalent for activatory (tri-methylation of histone H3 at lysine 4, H3K4me3) and inhibitory (tri-methylation of histone H3 at lysine 27, H3K27me3) histone modifications [2], with transcription being repressed primarily by Polycomb complexes catalyzing tri-methylation of H3K27 [3,4]. However, the chromatin structure of pluripotent cells is globally ‘open’ and more transcriptionally permissive [5,6], and has been recently suggested to be refractory to repression by Polycomb, relative to differentiated cells [7]. Moreover, in an induced ground pluripotent state [8], lineage-specification genes exhibit even lower expression and, paradoxically, reduced H3K27me3 [9]. In these conditions increased Pol II pausing is seen at these loci, which may be an alternative mechanism for maintenance of the transcriptional poised state.

Although recruitment of the Pol II machinery to the TSS is the most widely studied mode of transcriptional regulation, pausing of Pol II has recently emerged as a central step in this process [10]. The small nuclear non-coding RNA (ncRNA) *Rn7SK/7SK* has an important role in the regulation of transcriptional pausing [11-13], but its function in pluripotent cells has not been assessed. *7SK* is an abundant RNA of around 330 nucleotides, which is transcribed by Pol III and is highly conserved across jawed vertebrates [14]. *7SK* is present in a small nuclear ribonucleoprotein (snRNP) complex with proteins such as hexamethylene bis-acetamide inducible 1 mRNA (HEXIM) 1/2, La-related protein 7, and methylphosphate capping enzyme [12]. The 7SK snRNP has been shown to sequester positive transcription elongation factor b (P-TEFb), a kinase complex that phosphorylates Pol II, thereby preventing elongation [11,13,15,16]. Binding of the *7SK* RNA to HEXIM leads to a conformational change of this protein, facilitating its binding to and inactivation of the kinase domain of P-TEFb [12,17,18].

In this study, we investigated the role of *7SK* in mouse ESC transcription. We found that *7SK* not only regulates the transcription of a specific subset of genes with bivalent marks, but also of genes solely with active chromatin marks. Furthermore, *7SK* prevents widespread upstream divergent transcription and affects transcriptional termination of specific genes. Our study places the ncRNA *7SK* in a central position in the control of transcription in ESCs.

## Results

### *7SK* ncRNA is a gene-specific transcriptional repressor in ESCs

**Figure 1:**
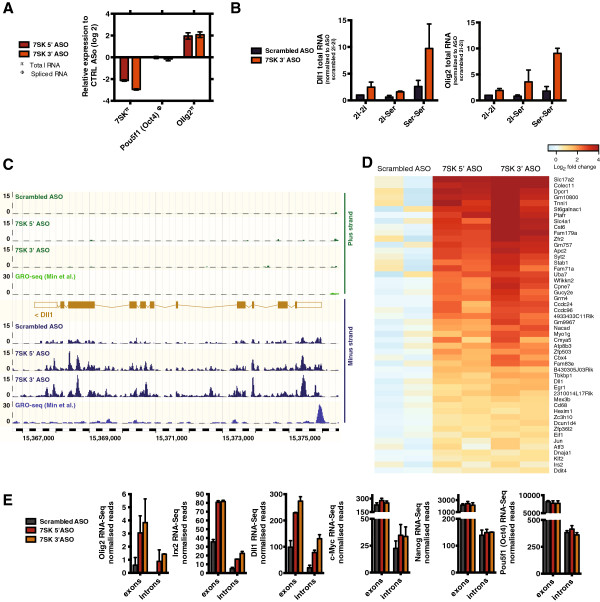
***7SK *ncRNA as a gene-specific transcriptional repressor in embryonic stem cells (ESCs). (A)** qRT-PCR analysis of *7SK* and *Olig2* total RNA (nascent and processed RNA), and *Pou5f1* (Oct4) mRNA 6 hours after nucleofection of ESCs with antisense oligonucleotides (ASOs) targeting the 5′ and 3′ segments of *7SK*, with green fluorescent protein (GFP) and scrambled ASOs as control. Error bars represent standard error of the mean (SEM) from 2 to 3 independent experiments. **(B)** Quantitative reverse transcription (qRT)-PCR analysis of *Dll1* and *Olig2* total RNA in ESCs 6 hours after nucleofection with *7SK* 3′ ASOs. ESCs were grown in serum (Ser-Ser) or 2i/LIF medium (2i-2i), or were switched from 2i/LIF to serum-containing media after nucleofection (2i-Ser). Error bars represent SEM from two independent experiments. **(C)** RNA sequencing (RNA-seq) read coverage at the *Dll1* locus. For this and all other genome browser images, read counts were normalized (see Materials and Methods), averaged over biological replicates, and visualized with Ensembl. The plus (green) and minus (blue) strand reads are displayed in separate tracks. **(D)** The 50 most significantly upregulated genes after *7SK* knockdown (that is, having the lowest *P*-values) were sorted by fold change. Color scale indicates expression relative to scrambled ASO mean (two biological replicates per ASO, assayed by RNA-seq). **(E)** Exonic and intronic normalized RNA-seq read counts for *Olig2*, *Irx2*, *Dll1*, *c-Myc*, *Nanog*, and *Pou5f1* (*Oct4*), averaged over replicates.

To investigate the role of *7SK* in the control of transcription in pluripotent cells, mouse ESCs were nucleofected with two distinct antisense oligonucleotides (ASOs) targeting segments near the 5′ [13] or 3′ ends of the *7SK* transcript. We observed a 70–85% knockdown of *7SK* RNA levels after 3 hours, which was sustained at 6 and 24 hours (Figure 1A; see Additional file 1: Figure S1). We tested the transcriptional effects on lineage-specification genes such as *Olig2* and Delta-like 1 (*Dll1*), which are expressed at very low levels in mouse ESCs, and found that levels of nascent and processed transcripts (hereafter referred to as ‘total RNA’) were rapidly increased upon *7SK* knockdown (Figure 1A,B; see Additional file 1: Figure S1). By contrast, pluripotency-associated genes, such as *Sox2* and *Pou5f1* (Oct4), were not affected (Figure 1A; see Additional file 1: Figure S1, and data not shown). We investigated whether *7SK* could mediate transcriptional repression of lineage-specification genes in ESCs in a naive ground pluripotent state, induced by switching from serum-containing medium to 2i/LIF, a defined medium containing inhibitors of the mitogen activated protein kinase/extracellular regulated kinase (MEK/ERK) and glycogen synthase kinase 3 (GSK3) pathways in combination with leukemia inhibitory factor [8]. We found that *7SK*-repressed genes such as *Dll1* and *Olig2* were indeed downregulated in 2i/LIF, whereas *7SK* levels remained unchanged (see Additional file 1: Figure S1). Moreover, *7SK* knockdown in ground-state conditions upregulated total RNA of *Dll1* and *Olig2* (Figure 1B), but not *Pou5f1* (Oct4) (see Additional file 1: Figure S1), to levels similar to those seen in ESCs cultured in the presence of serum. Nevertheless, we observed that transcriptional poising of lineage-specific genes by *7SK* in ESCs is more prominent in serum conditions (Figure 1B).

Our results suggested that *7SK* regulates the expression of lineage-specification genes in ESCs. In order to determine the genome-wide effects of *7SK*, we analyzed the transcriptome of ESCs grown in serum-containing media, after acute knockdown of *7SK* for 6 hours. For this purpose, we used strand-specific RNA sequencing (RNA-seq) targeting total RNA, without poly(A)^+^ selection, and after ribosomal RNA depletion (see Additional file 1: Figure S1). Although the majority of the annotated genes were not significantly affected by *7SK* knockdown, we found a cohort of 438 genes (including *Dll1* and *Nr4a2*) that were upregulated after *7SK* knockdown by both ASOs (Figure 1C, D; see Additional file 2: Figure S2) and 30 genes that were downregulated at a fold-change threshold of 1.5 and estimated false discovery rate below 5% (see Additional file 3: Table S1; see Additional file 4: Table S2). Gene Ontology (GO) analysis indicated that genes upregulated after *7SK* knockdown are highly enriched for those involved in transcription and (neural) development (see Additional file 2: Figure S2). Downregulated genes showed no enrichment, with an adjusted *P-*value of less than 0.01. RNA-seq data indicated increased transcriptional activity at upregulated genes throughout their loci, including at intronic regions (Figure 1C, E; see Additional file 5: Figure S3). Genes with significantly increased mRNA levels (exonic counts) showed a similar increase in intron expression, whereas non-regulated highly expressed genes such as *c-Myc*, *Nanog*, and *Pou5f1* (Oct4) did not present higher levels of intronic reads after *7SK* knockdown (Figure 1E; see Additional file 5: Figure S3). Thus, these results suggest that *7SK* represses the expression of nascent transcripts in specific loci, consistent with its function as a gene-specific transcriptional repressor.

### *7SK* knockdown is associated with failed transcriptional termination at specific loci

**Figure 2:**
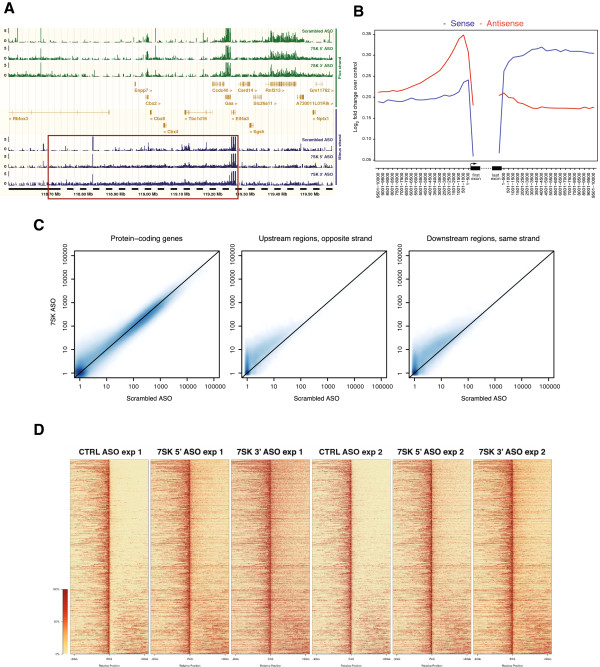
***7SK *knockdown is associated with failed transcriptional termination at specific loci. (A)** RNA sequencing (RNA-seq) read coverage plot showing that *7SK* knockdown results in increased transcription across an extensive region (box) downstream of *Eif4a3*, including *Cbx4*. The plus (green) and minus (blue) strand reads are displayed in separate tracks. **(B)** Mean change in RNA-seq read coverage around protein-coding genes after *7SK* knockdown. Log_2_ fold changes on the sense (blue) and antisense (red) strands were determined in 500 bp windows, and averaged over genes. **(C)** Density scatter plots of normalized read counts for protein-coding genes and surrounding regions. Counts from experiments in which ESCs were nucleofected with 5′ and 3′ *7SK* ASOs (*y*-axis) are plotted against counts for ESCs nucleofected with scrambled control ASOs (*x*-axis), to illustrate the overall change in expression levels after 7SK depletion. Color intensity indicates the density of data points. Read counts were normalized by the total number of mapped reads per sample (see Materials and Methods), incremented by a pseudocount of 1 to enable visualization on a logarithmic scale, and averaged over samples. **(D)** Heatmap of failed transcriptional termination after nucleofection of ESCs with *7SK* 5′ and 3′ ASOs. Each row represents a potential locus of failed transcriptional termination, centered at the 3′ end of the gene (polyadenylation site; PAS) and extending 100 kb upstream and downstream. Genes were ordered by first combining the normalized read distributions about the PAS for the six samples into a single vector for each gene, and are displayed in order from the highest average fold change (at the top) to the lowest.

Unexpectedly, we found increased transcription flanking several of these genes (for instance *Cbx4*, Figure 2A) and originating from the same strand, indicating broad genomic regions where transcriptional repression is mediated by *7SK*. Genome-wide analysis showed strong upregulation of transcription both upstream (antisense) and downstream (sense) of genes after *7SK* knockdown (Figure 2B, C; see Additional file 6: Figure S4). We identified 1,894 genes with increased downstream sense-strand read coverage after *7SK* knockdown (Figure 2D; see Additional file 7: Table S3), indicating continued production of transcripts downstream of polyadenylation sites (PASs). For the vast majority (86.2%) of these genes, transcription continued past the annotated end site for at least 1kb (in 48.7% of cases, for up to 10 kb) before reaching another gene. This downstream transcriptional activity often extended further from the initiating gene and across large chromosomal regions encompassing several other genes on the same strand (Figure 2). These regions spanned a total of 9170 genes, although they were not preferentially located in gene-rich areas (see Additional file 8: Figure S5). Notably, genes with failed transcriptional termination were not themselves upregulated in response to *7SK* knockdown (see Additional file 8: Figure S5), indicating a specific effect of this knockdown on the termination of transcription.

### *7SK* ncRNA directly represses a subset of genes with bivalent or active chromatin marks

**Figure 3:**
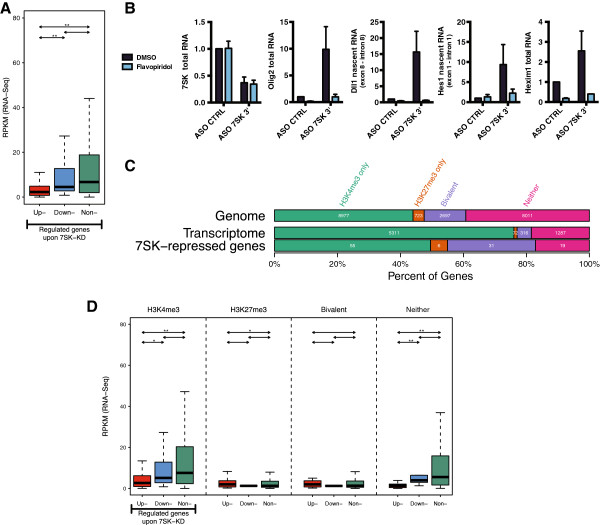
***7SK *ncRNA directly represses a subset of genes with bivalent or active chromatin marks in embryonic stem cells (ESCs), through a mechanism involving positive transcription elongation factor b (P-TEFb). (A)** Box plot of RNA sequencing (RNA-seq) gene expression values (reads per kilobase per million (RPKM); see Materials and Methods), averaged over the control antisense oligonucleotide (ASO) samples, for genes that were upregulated (left, red), downregulated (middle, blue) and not significantly altered (right, green) by *7SK* knockdown. Data are shown for the set of genes considered for differential expression analysis (see Materials and Methods). **(B)** Quantitative reverse transcription (qRT)-PCR analysis of *7SK, Olig2*, and *Hexim1* total RNAs, and for *Dll1* and *Hes1* nascent RNAs 6 hours after nucleofection of ESCs with scrambled *7SK* 3′ ASOs, in the presence or absence of flavopiridol. Error bars represent standard error of the mean (SEM) from two to three independent experiments. **(C)** Histone modification status in mouse ESCs [2] for all protein-coding and long intergenic non-coding RNA (lincRNA) genes larger than 1 kb (top), the subset expressed in ESCs (middle; RPKM > 5 in control ASO sample), and the subset directly repressed by *7SK* (bottom). Similar results were obtained when data were compared with those of Young *et al*. [79]**(D)** Box plots of gene expression values as in panel **(A)**, further stratified by chromatin mark status as in panel **(C)**. *P < 0.05, **P < 0.01; Kolmogorov-Smirnov test.

To identify genes subject to direct repression by *7SK,* while controlling for indirect transcriptional changes due to failed transcriptional termination at an upstream gene, we implemented a background-reduction filter. For each gene and sample, a background signal was estimated as the median read coverage (number of mapped reads per base pair) over five 2 kb regions at distances of 1 to 3, 3 to 5, 5 to 7, 7 to 9, and 9 to 11 kb upstream of the gene. Only reads mapped to the strand of the gene were counted. Segments of the 2 kb regions that coincided with exons of other genes annotated on the same strand were masked out, in order to base the background estimate on intronic and intergenic transcription only (for further description, please see Materials and Methods). Using this approach, we identified 122 genes that were under direct *7SK* repressive control (see Additional file 9: Table S4). Although pausing has been proposed to be associated with the tuning of expression of active genes [10,19], the level of expression of the genes repressed by *7SK* in ESCs was substantially lower than those unaffected by *7SK* knockdown (Figure 3A). GO analysis indicated that *7SK*-regulated genes are highly enriched for those involved in transcription, metabolic processes, and development/differentiation, highlighting the specificity of *7SK*-repression in ESCs (see Additional file 8: Figure S5). Most of the *7SK*-repressed genes (81.1%) were found to be occupied by transcriptionally engaged and elongation-competent Pol II at the TSS, as assessed by comparing our data with a global run-on sequencing (GRO-seq) dataset from mouse ESCs [1] (*P* = 1.34 × 10^-21^, Fisher’s exact test, compared with 53.7% in the genome, 10989 out of 20465 genes and lincRNAs). In accordance with this, treatment with flavopiridol, an inhibitor of positive transcription elongation factor b (P-TEFb) abolished the increase in nascent transcript levels by *7SK* knockdown (Figure 3B). There was a robust enrichment for bivalent genes [2] among those repressed by *7SK* (27.9%), in relation to the ESC transcriptome (4.5%, *P* = 3.44 × 10^-9^, Fisher’s exact test) (Figure 3C). Interestingly, 49.5% of the genes repressed by *7SK* were marked with H3K4me3 in the absence of H3K27me3 (Figure 3C). As with all *7SK*-repressed genes, these genes exhibited low levels of expression in ESCs (Figure 3D), suggesting that *7SK* provides a novel mechanism of repression for these genes in pluripotent cells, distinct from the established mechanism involving Polycomb activity.

### *7SK* ncRNA represses upstream divergent transcription

**Figure 4:**
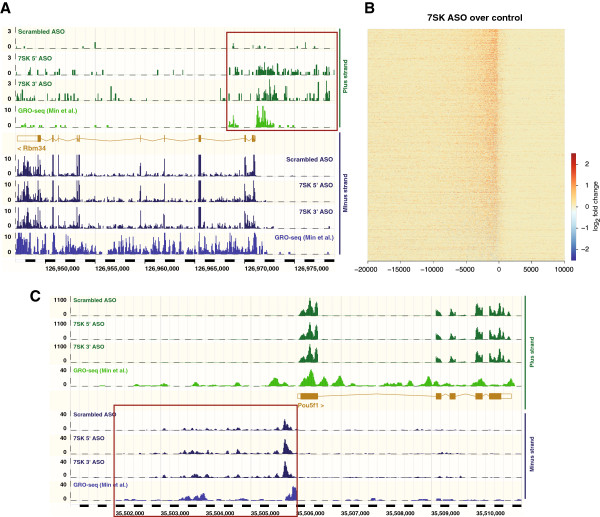
***7SK *represses upstream divergent transcription. (A)** Ensembl genome browser image of the *Rbm34* locus, showing normalized RNA sequencing (RNA-seq) read coverage (mean of two biological replicates) for ESCs nucleofected with scrambled control antisense oligonucleotides (ASOs) or *7SK* ASOs. Published global run-on sequencing (GRO-seq) data for ESCs [1] indicated occupancy of transcriptionally engaged Pol II. Purple box highlights upstream divergent RNA (udRNAs). The plus (green) and minus (blue) strand reads are displayed in separate tracks. **(B)** Change in udRNA expression after *7SK* knockdown for all 2676 genes (rows) with a detected udRNA. Colors indicate fold change on the antisense strand in 50 bp windows around the transcription start site (TSS). **(C)** RNA-seq and GRO-seq read coverage at the *Pou5f1 (Oct4)* locus. The udRNA region is highlighted in purple box. Note that different scales are displayed for plus/minus strand and GRO-seq tracks in panels **(A)** and **(C)**.

Interestingly, as indicated above, we found widespread transcription upstream of the TSSs of annotated genes in the antisense/divergent orientation (Figure 2B, C). Applying conservative criteria to exclude loci where such divergent transcription might be confounded with reads from neighboring protein-coding genes (see Materials and Methods), we identified 2676 genes with strong evidence of divergent transcription within 5 kb upstream of annotated TSSs (Figure 4; see Additional file 10: Table S5). We refer to these transcripts as upstream divergent RNAs (udRNAs), and note that such RNAs are also expressed in human ESCs [20] (see Additional file 8: Figure S5). We found that 22.7% of the udRNAs overlapped with divergent TSS-associated RNAs previously detected in mouse (see Additional file 11: Figure S6). RNA-seq read coverage indicated that these udRNAs could extend several kilobases upstream of the TSS (Figure 2B; Figure 4).

**Figure 5:**
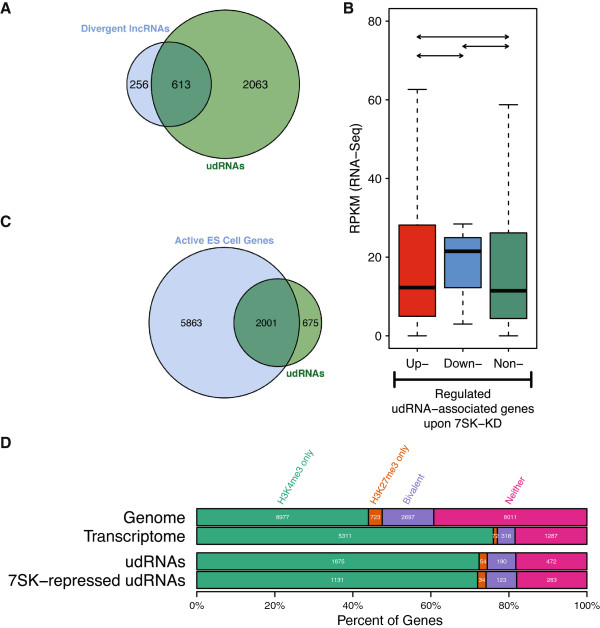
**udRNAs overlap with divergent lncRNAs and are associated with transcriptionally active genes. (A)** Venn diagram showing that 70.5% of genes with an associated divergent lncRNA in the upstream region [21] overlap with udRNAs. **(B)** Box plot of RNA-seq expression values, averaged over the control ASO samples, of genes associated with udRNAs, stratified by change in expression after *7SK* knockdown (see also Figure 3A). **(C)** Venn diagram showing the proportion of expressed genes (RPKM > 5 in control ASO samples) found to be associated with udRNAs. **(D)** Overlap between genes and udRNAs with genomic regions enriched for histone modifications H3K4me3, H3K27me3 or both (bivalent) in mouse ESCs [2] (see also Figure 3C). Similar results were obtained when comparing with Young *et al*. [79]. *P < 0.05, **P < 0.01; Kolmogorov-Smirnov test.

A recent study identified numerous long ncRNAs (lncRNAs) transcribed from active promoters of protein-coding genes in mouse ESCs in the divergent orientation [21]. Of the loci searched for udRNAs here, 869 were found to encode such upstream divergent lncRNAs, and we detected udRNAs at 613 of those (70.5%; Figure 5A). Moreover, we also observed a general trend for long intergenic ncRNAs (lincRNAs) to be upregulated after *7SK* knockdown in mouse ESCs. For the 2,057 lincRNAs annotated in the Ensembl database, expression levels were increased by 18% on average (geometric mean for background-adjusted data) after *7SK* knockdown (see Additional file 3: Table S1; see Additional file 4: Table S2; see Additional file 9: Table S4). This is a larger increase than expected for any group of genes (*P* < 10^-6^, randomization test).

Quantitative expression analysis showed that the majority of detected udRNAs were upregulated by *7SK* knockdown (Figure 2B; Figure 4B), with 94.5% displaying a positive fold change and 60.5% upregulated more than two-fold, again consistent with the repressor role of *7SK*. Of the udRNAs overlapping with divergent lncRNAs [21], 44.69% (274 of 613) were upregulated by more than two-fold after *7SK* knockdown (see Additional file 11: Figure S6). We found, in contrast to the *7SK*-repressed lineage-specific genes, that genes associated with *7SK*-repressed udRNAs were transcriptionally active (Figure 5B). Indeed, at least a quarter of the active genes in ESCs were found to be associated with udRNA expression (Figure 5C), and 71.9% of the genes associated with *7SK*-repressed udRNAs were marked with H3K4me3 alone (Figure 5D).

**Figure 6:**
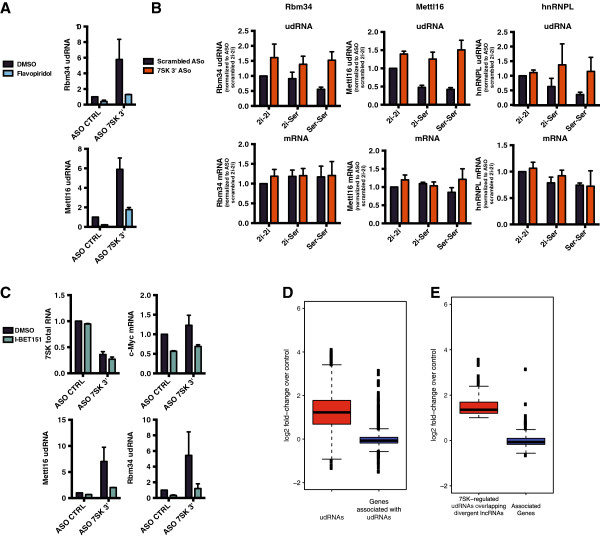
***7SK *represses upstream divergent RNAs (udRNAs) and long non-coding RNA (lncRNAs) but not their associated transcriptionally active genes, and positive transcription elongation factor b (P-TEFb) is involved in udRNA transcription. (A)** Quantitative reverse transcription (qRT)-PCR analysis of *Rbm34* and *Mettl16* udRNAs 6 hours after nucleofection of embryonic stem cell (ESCs) with scrambled or *7SK* 3′ antisense oligonucleotides (ASOs), in the presence or absence of flavopiridol. Error bars represent standard error of the mean (SEM) from two independent experiments. **(B)** qRT-PCR analysis of udRNAs adjacent to *Rbm34*, *hnRNPL*, and *Mett1l6*, and corresponding mRNAs 6 hours after nucleofection of mouse ESCs with control ASOs or ASOs targeting *7SK*. ESCs were grown in serum (Ser-Ser) or 2i/LIF media (2i-2i), or switched from 2i/LIF to serum media after nucleofection (2i-Ser). SEM from two to three independent experiments. **(C)** qRT-PCR analysis of *7SK* total RNA, *c-Myc* spliced mRNA, and *Rbm34* and *Mett1l6* udRNAs, 6 hours after nucleofection of ESCs with scrambled of *7SK* 3′ ASOs, in the presence or absence of I-BET151. Error bars represent SEM from two to three independent experiments. **(D)** Box plot depicting log_2_ fold changes measured by RNA sequencing (RNA-seq) after *7SK* knockdown of udRNAs and their associated genes in mouse ESCs. **(E)** Box plot depicting log_2_ fold changes measured by RNA-seq after *7SK* knockdown in mouse ESCs of *7SK*-regulated udRNAs overlapping divergent long intergenic non-coding RNAs (lincRNAs) and their associated genes.

We found a striking overlap between udRNA RNA-seq reads and GRO-seq data, which also identified Pol II engaged upstream of annotated genes in mouse ESCs [1] (Figure 4A,C). Overall, 88.5% of *7SK*-repressed udRNAs were found to have transcriptionally engaged Pol II. The role of *7SK* in transcriptional pausing has been previously shown to involve sequestering the P-TEFb kinase, thereby preventing Pol II phosphorylation at serine 2 [12]. Treatment with the P-TEFb inhibitor flavopiridol abolished the increase in udRNA levels induced by *7SK* knockdown (Figure 6A), confirming that Pol II can initiate and elongate transcription at these loci. Similar results (Figure 6C) were obtained after treatment with I-BET151 [22], an inhibitor of bromo and extra terminal (BET) bromodomain proteins, which recruit P-TEFb to acetylated histones and lead to activation of transcription [22,23]. Similar to *7SK*-repressed genes, repression of udRNA transcription by *7SK* was more pronounced in serum-containing media than in 2i/LIF (Figure 6B). Genes with *7SK*-regulated udRNAs were associated with diverse cellular processes (see Additional file 12: Table S6). Strikingly, these genes were mostly unaffected by *7SK* knockdown (Figure 6B,D; see Additional file 10: Table S5). A similar pattern was seen with *7SK*-regulated udRNAs overlapping divergent lncRNAs (Figure 6E), suggesting that *7SK* prevents the coordinated expression of this subset of lncRNA/mRNA gene pairs.

## Discussion

**Figure 7:**
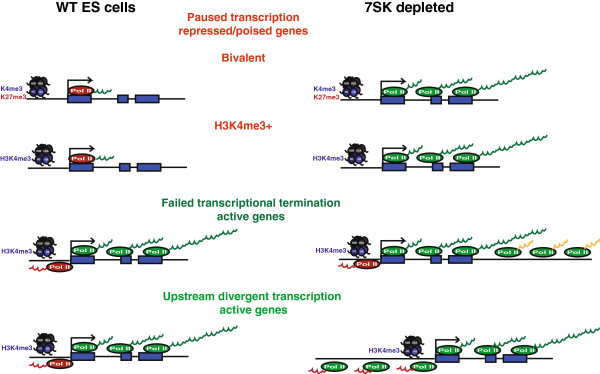
**The non-coding RNA (ncRNA) *7SK *has a central role in controlling transcription in embryonic stem cells.***7SK* is required for the repression of genes that are silent or expressed at a low level. Widespread failed transcriptional termination was also seen after *7SK* knockdown. *7SK* is a major regulator of transcriptional directionality, by preventing the transcription of upstream divergent RNAs (udRNAs).

Several classes of regulatory RNAs are emerging as important regulators of gene expression, cell-fate determination, and development [24-31]. ncRNAs, including microRNAs [32] and lncRNAs [26], have been recently implicated in the control of pluripotency. Our study shows that a single ncRNA, *7SK*, controls different aspects of transcription at specific loci in ESCs (Figure 7). *7SK* represses a very specific cohort of genes, including several that are pivotal in lineage specification. A substantial proportion of the genes whose expression levels increased after *7SK* knockdown do not have bivalent chromatin marks, but rather have H3K4me3, indicating that *7SK* may inhibit transcription at a novel subset of gene loci where Polycomb repression is not operational. These results are consistent with recent findings that pluripotent chromatin in general is refractory to repression by Polycomb [7], and that H3K27me3 is reduced at genes whose expression is lower in an induced ground pluripotent state [9]. However, although elongation has been characterized as a major regulator of transcription of active genes in ESCs [9,19], our data suggest that *7SK* is not required for the fine-tuning of transcription of these genes.

P-TEFb has been shown to regulate transcription and cell fate during embryonic development in *Caenorhabditis elegans*[33], *Drosophila*[34] and zebrafish [35], and *7SK* expression is increased upon ESC differentiation into neural (neuronal and glial) lineages [30]. Therefore, we extended our analysis to neural committed cell types: neural stem cells (NSCs) [36] and oligodendrocyte precursor cells (OPCs) [37]. In contrast to ESCs, we did not observe effects on the expression of *Olig2* total RNA, which is expressed in higher levels in these cells, after *7SK* knockdown (see Additional file 13: Figure S7). Other genes expressed at higher levels in these cells, such as *Sox9* (NSCs) and *Sox2* (OPCs), were also not affected by *7SK*. However, there was an increase in nascent transcript levels for specification genes such as *Nr4a2, Hes1*, and *Irx2* after *7SK* knockdown in NSCs (see Additional file 13: Figure S7). We found a similar increase in nascent transcription of *Dll1* and of genes involved in oligodendrocyte differentiation, such as the genes encoding for myelin basic protein (*Mbp*) and 2′,3′-cyclic-nucleotide 3′-phosphodiesterase (*Cnp*) after *7SK* knockdown in OPCs (see Additional file 13: Figure S7). These results indicate that the repression of lineage specification/differentiation genes by *7SK* is maintained in neural lineage cell populations. In a manner analogous to Polycomb activity [38], *7SK* repression appears to affect different cohorts of genes depending on the transcriptional and developmental state of the cell.

These results indicate that *7SK* plays an important role in the control of transcription of lineage specification/differentiation genes in stem/progenitor cells. It has been previously shown that disruption of the *7SK* snRNP is rapidly compensated for by the increased expression of another component of the complex, HEXIM1 [39]. We found upregulation of *Hexim1* total RNA in both ESCs (Figure 1D; see Additional file 11: Figure S6) and in OPCs (see Additional file 13: Figure S7), suggesting a similar feedback mechanism to control P-TEFb availability after *7SK* depletion.

This study also identified two completely novel functions of *7SK* in preventing downstream (sense) and upstream (antisense) transcription, at specific and distinct active loci. The increased downstream sense transcription seen after *7SK* knockdown might be associated with failed transcriptional termination by Pol II [40] or lengthening of 3′ untranslated regions (UTRs) [41]. The latter appears to be considerably more frequent in neural lineages than in ESCs [41]. *7SK* might thus be a key component in restricting 3′ UTR length in certain cell types, including ESCs, through a mechanism less active in differentiated neural cell types.

Widespread upstream divergent antisense transcription has previously been described in several species [21,42-49]. In ESCs, this phenomenon was primarily found to produce short RNAs (20 to 90 nucleotides) [50]. Recent studies indicated that some of these transcripts can extend up to 1,100 kb [51], and that a majority of lncRNAs expressed in mouse ESCs derive from bidirectional transcription at active gene promoters [21,52]. The results here extend these findings, identifying novel loci of divergent upstream transcription, extending over several kb upstream of the TSS. They also indicate that *7SK* plays a role in the expression of a subset of these divergent lncRNAs. lncRNA/mRNA gene pairs have been reported to show coordinated expression after differentiation of ESCs [21]. However, our data indicate that 7SK represses divergent lncRNA expression specifically, rather than that of the associated mRNA, implying that neighboring lncRNA and coding genes can be regulated through different mechanisms. Moreover, the degradation of divergent antisense RNAs can be mediated by the exosome [42,46,49,51], and our results suggest that this might be complemented by the activity of *7SK* in preventing divergent upstream transcription. *7SK* knockdown also led to upregulation of udRNAs in NSCs and OPCs (see Additional file 13: Figure S7), suggesting that repression of antisense transcription is a general function of *7SK*.

P-TEFb kinase complex is involved in the functions of *7SK* described here, as treatment with the P-TEFb inhibitor flavopiridol (Figure 3, Figure 6) [51] suppressed the transcription of poised genes and udRNAs after *7SK* knockdown. In addition, I-BET151 prevented the upregulation of udRNAs by *7SK* knockdown (Figure 6), indicating that bromodomain-containing protein 4 (BRD4)-mediated P-TEFb recruitment is involved in the *7SK* upregulation of udRNAs. This effect was not as prominent for *Dll1* (see Additional file 11: Figure S6), which might reflect an alternative role of BRD4 in the association of P-TEFb with the inactive *7SK* complex [39,53], rather than inhibition of the recruitment of P-TEFb to the chromatin. Alternative and/or complementary mechanisms to P-TEFb are also likely to be required for *7SK*-mediated repression. For instance, divergent transcription and failed termination, which are both affected by *7SK*, can be inhibited via gene looping [54,55]. The polyadenylation complex factor Ssu72, which is a phosphatase of Pol II, has been shown to be pivotal to these processes in *Saccharomyces cerevisiae*[54,55]. Interestingly, transcriptional termination and elongation in HIV can also be regulated by a regulatory region of the HIV RNA genome, *TAR*[56], which has some structural similarities with *7SK*[12], and has been proposed to displace *7SK* to enable trans-activation of HIV genes [57]. While this paper was under revision, Sharp and colleagues published a paper describing a novel regulatory system that controls promoter directionality, based on enrichment of canonical polyadenylation signals and Pol II termination upstream of genes, and enrichment of U1 small nuclear RNA (snRNA) sites downstream of the TSS, preventing premature termination of the sense RNA [58]. Interestingly, SR proteins, which interact with the U1 small ribonucleoprotein, have recently been shown to be components of the *7SK* complex [59]. These mechanisms might be operational in the repression of upstream transcription and control of termination by *7SK*.

Most of the *7SK* snRNP sequesters P-TEFb in an inactive complex in the nucleoplasm [15-17,23,60,61], and in nuclear speckles [13]. *7SK* knockdown leads to reorganization of proteins associated with interchromatin granule clusters, including SR proteins [13], and these events could be involved in the transcriptional events we found here. Nevertheless, our results also indicate that *7SK* repression operates at specific loci in the genome, and thus, specific recruitment mechanisms may be in place. Indeed, it has been recently shown that *7SK* ncRNA is a chromatin component [62], and transiently associates with repressed genes [13]. Moreover, the *7SK* snRNP component HEXIM1 can be located at active gene promoters in mouse embryonic fibroblasts [59]. Chromatin-modifying enzymes, some of which have been shown to interact with ncRNAs in mouse ESCs [26] and/or transcription factors, are also among the candidates for potentially targeting *7SK* to specific loci to act as gene-specific transcriptional repressor. *7SK* has been recently shown to interact with the transcription factor high-mobility group A1 (HMGA1) and to modulate its transcriptional activity in both P-TEFb-dependent and P-TEFb-independent manners [63-65]. The transcription factor c-Myc has also been shown to recruit P-TEFb to active genes in mouse ESCs, and to modulate transcriptional elongation [19]. Interestingly, c-Myc expression is decreased in ESCs cultured in 2i/LIF, but promotes elongation only of a small subset of genes in ESCs grown in serum-containing media [9], which implies that there are other unknown factors regulating the promoter-specific poising. P-TEFb can also be recruited by the super elongation complex (SEC) to paused active genes in mouse ESCs, while after differentiation, SEC is recruited to activated developmental genes [66]. Further investigation will determine if some of these molecules contribute to the mechanism by which *7SK* regulates the diverse transcriptional outcomes identified here, and whether these are related or independent events.

## Conclusion

Our study reveals that the ncRNA *7SK* acts as a repressor of a cohort of poised genes in ESCs, and unexpectedly modulates several other processes, including upstream (antisense) and downstream (sense) transcription. The actions of *7SK*, although widespread, primarily affect specific sets of genes, indicating that mechanisms for targeting *7SK* to discrete genomic loci might be in place.

## Materials and methods

### Cell culture

Oct4-GiP ESC [67] were maintained in ES media consisting of Glasgow Minimum Essential Medium (GMEM) supplemented with 10% fetal calf serum for ESCs (Biosera, Boussen, France), 0.1 mmol/L non-essential amino acids, 2 mmol/l L-Glutamine, 1 mmol/l sodium pyruvate, 0.1 mmol/l β-mercaptoethanol, 1x penicillin/streptomycin and 10^6^ units/L LIF (ESGRO, MilliporeCorp., Billerica, MA, USA). Alternatively, cells were grown in 2i/LIF media, based on GMEM and containing 10% Knock-Out Serum Replacement (Life Technologies Corp., Carlsbad, CA, USA), 1% fetal calf serum for ESCs (Biosera or Sigma-Aldrich (St Louis, MO, USA)), 0.1 mmol/l non-essential amino acids, 2 mmol/l L-glutamine, 1mmol/l sodium pyruvate, 0.1 mmol/l beta-mercaptoethanol, 1 μmol/l PD0325901 (AxonMedChem, Groningen, The Netherlands), 3 μmol/l CHIR99021 (AxonMedChem), 1x penicillin/streptomycin, and 10^6^ units/L LIF (ESGRO; Millipore). In addition, 1 μg/ml puromycin was added to ES Oct4-GIP cultures during expansion. NSO4G NSCs [36] were grown in RHB-A medium (Stem Cell Sciences, Cambridge, UK), supplemented with penicillin/streptomycin and 10 ng/ml basic fibroblast growth factor and epidermal growth factor (PeproTech, Rocky Hill, NJ, USA). ES Oct4-GIP and NSO4G cells were cultured in plates coated with 0.1% gelatin (Sigma-Aldrich). Oli-neu OPCs [37] were cultured in plates coated with 0.01% poly-L-lysine (Sigma-Aldrich) and grown in Sato media (with 340 ng/ml T3 and 400 ng/ml L-thyroxine; Sigma-Aldrich) supplemented with 1% horse serum (Invitrogen) as previously described [37]). OPCs were lipofected with 100 nmol/l ASOs using Lipofectamine 2000 (Invitrogen). Opti-MEM I reduced serum medium was used to prepare the complexes. Cells were incubated with the complexes for 4 hours in DMEM (Invitrogen Corp., Carlsbad, CA, USA) before replacing media with the original. Flavopiridol and I-BET151 were used at 500 nmol/l for 6 hours. ASOs (1,000 pmol) were nucleofected into mouse ESCs using the Mouse ES Cell Nucleofector Kit (program A23; Lonza AG, Basel, Switzerland). NSO4G cells were transfected with 400 pmol ASOs using the Cell Line Nuclefector Kit V (program T20; Lonza AG). After nucleofection, ESCs/NSCs were plated into gelatin-coated wells, and collected with Qiazol (Qiagen Inc., Valencia, CA, USA) at the indicated time points for RNA extraction. ASOs (Table S7) were synthesized by Integrated DNA Technologies (Coralville, IA, USA). Total RNA was isolated from ESCs and NSO4G using the miRNeasy Extraction Kit (Qiagen), with in-column DNAse treatment.

### qRT-PCR

Genbank and Ensembl cDNA sequences were used to design gene-specific primers in Primer 3 [68] or in the Universal ProbeLibrary Assay Design Center (Roche Applied Science, Indianapolis, IN, USA). The specificity of the PCR primers was determined by *in silico* PCR (UCSC Genome Browser) and Primer-BLAST (NCBI) programs. PCR primers (see Additional file 14: Table S7. were synthesized by Sigma-Aldrich. DNase-treated total RNA was reverse-transcribed with random primers for 1 hour, using the High-Capacity cDNA Reverse Transcription Kit; Applied Biosystems, Foster City, CA, USA), in accordance with the manufacturer’s instructions. Each sample was equally divided into two aliquots: a cDNA reaction tube,and a negative control tube without reverse transcriptase (RT-negative). Before qPCR analysis, both cDNA and RT-negative samples were diluted 5 or 10 times, with DNase/RNase-free distilled water (Ambion Inc., Austin, TX, USA). qPCR reactions were performed in duplicate or triplicate for each sample. Each individual PCR was carried out with a final volume of 10 to 20 μl and 2.5 to 5 μl of diluted cDNA. The RT-negative setup was run for a few samples in each run to discount genomic DNA amplification. The Fast SYBR Green Master Mix (Applied Biosystems) was used in accordance with the manufacturer's instructions. A melting curve was obtained for each PCR product after each run, in order to confirm that the SYBR Green signal corresponded to a unique and specific amplicon. Random PCR products were also run in a 2 to 3% agarose gel to verify the size of the amplicon. Standard curves were generated for each qPCR run,and were obtained by using serial three-fold dilutions of a sample containing the sequence of interest. The data were used to convert *C*_*t*_ values to arbitrary units of the initial template for a given sample. Expression levels in all experiments were then obtained by dividing this quantity by the value of the housekeeping gene TATA-binding protein (TBP) in the *7SK* knockdown experiments (because TBP is not affected by *7SK* knockdown; data not shown) or 18S ribosomal RNA in the flavopiridol and I-BET151 experiments (18S expression is not affected by flavopiridol or I-BET151, whereas TBP expression is affected by flavopiridol, but not by I-BET151; data not shown). Alternatively, the ^ΔΔ^*C*_*t*_ method was used.

### Strand-specific RNA-seq

Total RNA was depleted from ribosomal RNA with the Low Input Ribo-Zero™ rRNA Removal Kit (Epicentre Biotechnologies, Madison, WI, USA). No poly(A)^+^ selection was performed. Total RNA was then fragmented with RNA fragmentation reagent (Ambion), purified using the RNeasy MinElute Kit (Qiagen), and treated with alkaline phosphatase (New England Biolabs, Beverly, MA, USA) for 30 minutes at 37°C. The 5′ dephosphorylated RNA was then treated with T4 polynucleotide kinase (New England Biolobs) for 60 minutes at 37°C. The resulting RNA (5′ mono-phosphoryl and 3′ hydroxyl) was purified using the RNeasy MinElute Kit (Qiagen), and ligated with RNA 3′ and 5′ adapters, using the TruSeq Small RNA Sample Preparation Guide (Illumina Inc., San Diego, CA, USA) in accordance with the manufacturer’s instructions. Indexes 1 to 6 were used for PCR amplification. Libraries were quantified by Bioanalyzer (Agilent Technologies Inc., Wilmington, DE, USA) or absolute qPCR with a KAPA Library Quantification ABI Prism Kit (Kapa Biosystems Inc., Woburn, MA, USA and Applied Biosystems), and sequenced (50 nt single-end reads) on the HiSeq 2000 (Illumina).

### RNA-seq data processing and expression analysis

Sequence reads were processed to remove any trailing 3′-adapter sequence using Reaper (version 12–048) [69,70] with the following options: -3p-global 12/1/0/2 -3p-prefix 12/1/0/2 -3p-head-to-tail 1. Reads shorter than 20 nt after trimming were discarded. The remaining sequences were aligned to mouse genome assembly NCBIM37 (mm9) using GSNAP version 2012-04-21 [71]. GSNAP options were set to require 95% similarity and disable partial alignments (−m 0.05 --terminal-threshold = 100 --trim-mismatch-score = 0). To enhance alignment accuracy, GSNAP was provided with known splice sites from Ensembl 66 [72] and the RefSeq Genes and UCSC Genes tracks from the UCSC Genome Browser database [73]. Reads that coincided with ribosomal RNA genes from Ensembl or ribosomal repeats in the UCSC Genome Browser RepeatMasker track were excluded.

Expression levels were estimated for Ensembl genes by summing the counts of uniquely mapped reads, requiring that at least half the alignment overlap annotated exon sequence. This criterion was designed to retain exonic reads in cases where partial exons were annotated or reads were suboptimally aligned at exon boundaries (however, we noted that nearly identical expression values were obtained if 100% exon overlap was required; data not shown). For comparisons among genes, the read counts were normalized by exon model length and the total number of reads mapped to genes, to give reads per kilobase of exon model per million mapped reads (RPKM) [74]. Genes were classified as expressed if the mean of the control sample RPKMs was greater than 5.

For analysis of changes in gene expression after *7SK* knockdown, read counts were normalized to be comparable across samples using the trimmed mean of M-values (TMM) method implemented in the Bioconductor package edgeR [75,76]. We obtained very similar results with the alternative normalization method proposed by Anders and Huber [77]. To estimate expression fold change for regions upstream and downstream of genes, read counts for these regions were processed as the counts for genes: only uniquely mapped reads were considered, and normalization was carried out using the scaling factors determined for annotated genes by the TMM method. The same scaling factors were also applied for visualization of read coverage along the genome.

To verify that the observed increase in expression around genes could be observed independent of the use of gene annotation in the normalization, we additionally analyzed changes in distributions of reads after scaling raw counts so that the total number of mapped reads was identical between libraries. Specifically, read counts were divided by the total number of mapped reads per sample, and multiplied by the mean number of mapped reads across samples. The results of this analysis are shown in Figure 2C and confirmed trends observed with TMM normalization (see Additional file 6: Figure S4).

Differentially expressed genes were identified with the generalized linear model functions in edgeR, using a design matrix with two explanatory variables: antisense oligo type (anti-*7SK* or scrambled control) and experiment batch (1 or 2). To conservatively rule out off-target effects, model fitting and calling of differentially expressed genes were performed separately for each of the two *7SK* ASOs, and the results intersected. When testing each *7SK* ASO, genes with minimal evidence of expression were excluded by requiring a read count exceeding one read per million exonic reads in at least two samples. For all fold-change estimates, TMM-normalized read counts were incremented by a pseudocount of 1.

To identify genes with altered expression after *7SK* knockdown while controlling for failed termination of upstream genes, read counts were adjusted by subtracting an estimate of local background transcription. For each gene and sample, a background signal was estimated as the median read coverage (number of mapped reads per base pair) over five 2 kb regions at distances of 1 to 3, 3 to 5, 5 to 7, 7 to 9, and 9 to 11 kb upstream of the gene. Only reads mapped to the strand of the gene were counted. Segments of the 2 kb regions that coincided with exons of other genes annotated on the same strand were masked out, in order to base the background estimate on intronic and intergenic transcription only. Background estimates were scaled to account for the difference in size between the regions where background was measured and the exonic size of the gene. Expression values below the background were set to zero. Thus, for each gene *i,* the background-adjusted read count was computed as:

$$\max\;\left(0,g_{i}-l_{i}\times \underset{j}{\mathrm{median}}\left(\frac{a_{\mathrm{ij}}}{b_{\mathrm{ij}}}\right)\right)$$

where *g*_*i*_ is the unadjusted read count, *l*_*i*_ is the total exonic size of the gene, and *a*_*ij*_ and *b*_*ij*_ are the read counts and size (after masking exons) for the five associated regions (*j* = 1, 2, …, 5), from which the background signal was estimated.

### Detection of udRNA transcriptional units

The search for udRNAs was conducted using RNA-seq data for an equal number of control and knockdown samples to avoid introducing a bias towards udRNAs preferentially expressed in either condition. For the results described above, the *7SK* 5′ ASO data were omitted, thus leaving two biological replicates each for the scrambled ASO and the *7SK* 3′ ASO. Intergenic regions between closely spaced (<10 kb) and divergently oriented protein-coding genes were excluded from consideration, in order not to confound the udRNA reads with those from coding genes. For the remaining protein-coding genes, the 5 kb region immediately upstream was examined. This limit was motivated by a genome-wide trend for increased upstream transcription within 5 kb, after *7SK* knockdown (Figure 2B). Upstream regions were considered putative udRNA transcriptional units if there was a normalized count of at least 10 uniquely mapped reads on the opposite strand relative to the coding gene in any of the four RNA-seq samples. We regard this threshold as conservative, because the trend for increased transcription in upstream regions was apparent at lower read counts (see Additional file 11: Figure S6). It should be noted that the 5′ ASO data were only excluded for detection of putative udRNA regions. All RNA-seq data were used in the further analysis of those regions, such as calculation of fold change between knockdown and control conditions. Equivalent results were obtained when the 3′ ASO data were excluded instead (see Additional file 11: Figure S6), and the upregulation of udRNAs in all knockdown samples was evident (see Additional file 6: Figure S4).

An additional criterion was applied to distinguish udRNAs from failed termination regions extending across promoters (we found that some promoters exhibited antisense transcription, due to apparent failed termination of a downstream gene on the opposite strand; Figure 2A). For this purpose, read coverage at putative udRNA regions were compared to estimates of background transcription in a manner similar to the background adjustment described in the preceding section on gene expression analysis. For each gene, antisense read coverage was determined over five 2 kb regions at distances of 1 to 3, 3 to 5, 5 to 7, 7 to 9, and 9 to 11 kb downstream of the final TSS. Segments of these 2 kb regions that coincided with exons annotated on the opposite strand relative to the gene were masked out, in order to base the background estimate on intronic and intergenic transcription only. udRNA regions were required to have a read coverage at least two-fold greater than each of the five background regions (in at least one of the four RNA-seq samples considered). Thus, for each gene *i,* the threshold for normalized udRNA read count was computed as:

$$\max\left(10,2\times \;5000\times \max_{j}\left(\frac{c_{\mathrm{ij}}}{d_{\mathrm{ij}}}\right)\right)$$

where 5000 corresponds to the size of the udRNA region in base pairs, and *c*_*ij*_ and *d*_*ij*_ are the read counts and size (after masking exons) for the five associated regions (*j* = 1, 2, …, 5) from which the background signal was estimated.

### Overlap with known features

The level of overlap between known features and transcript regions was calculated using the intersectBed function from the bedTools package [78]. To avoid the likelihood of false-positive overlaps biasing the results, we limited our analysis to protein-coding genes and lincRNAs greater than 1 kb in length. Promoters were defined as the region 5 kb upstream and 1 kb downstream from the TSS, which were interrogated for the presence of known H3K4me3-enriched and/or H3K27me3-enriched sites [2,79], TSS-associated RNAs [43] and regions of engaged Pol II [1]. If necessary, feature coordinates were mapped to mm9 using the liftOver utility available from the UCSC Genome Browser website [80]. Transcripts were defined as having the feature if an overlap of at least one base was detected between the feature coordinates and the gene region coordinates. *P*-values for the enrichment of these genomic features in *7SK*-responsive genes were calculated using Fisher's exact test on the 2 × 2 contingency table.

For divergent lncRNA comparisons, we took the list of 1,667 divergent lncRNAs identified in murine ESCs by Sigova *et al*. [21], and compared these against the 1 kb region upstream of the TSSs of the 17,984 genes considered in our analysis. Any gene where this region intersected a divergent lncRNA on the opposite strand was considered to be associated with divergent lncRNA transcription. This resulted in 869 divergent lncRNA genes, which were compared with the 2,676 genes that had an associated udRNA identified in the 1 kb upstream region.

### Identification of genes with failed transcriptional termination

Each gene was subdivided into 100 regions of equal length, and the normalized read density (number of reads per base, normalized as previously described) was calculated for each bin for each sample. The 100 kb regions immediately upstream and downstream of the gene were also segmented into 500 bins of 200 bases each, and the normalized read density was computed. For each gene, regions of enrichment upstream of the TSS or downstream of the PAS were identified by searching for contiguous bins showing a minimum read density of 0.005 (corresponding to an average normalized read count of 1 within the 200 bp bin) within a sliding window of 10 bins. The normalized read count within these regions was determined, and all read counts were thresholded to a minimum of 1 to circumvent problems with subsequent fold-change analysis. The log_2_ fold change between the mean of each of the *7SK* knockdown sample pairs (*7SK* 5′ ASO and *7SK* 3′ ASO) and the control sample pairs was calculated. All genes showing a downstream region greater than 1 kb in size with a fold change greater than 1.5 were considered potential candidates for failed transcriptional termination, and were interrogated to identify further candidates within 100 kb upstream, which might represent the initiating locus. Candidate genes were defined as those actively transcribed, showing no evidence of upstream candidates (and so are likely themselves to be the initiating locus), and with a downstream region of enrichment greater than 3 kb.

### Identification of extent of downstream divergent transcription

For candidate genes where failed transcriptional termination may originate, the read distribution in 200 bp bins over a 1 Mb window upstream and downstream of the PAS was calculated using the Repitools [81] package in R. Genes were ordered by first combining the normalized read distributions about the PAS for the six samples into a single vector for each gene, and are displayed from the highest average fold change (at the top) to the lowest average fold change. We identified accurate estimates for the size of the failed termination region by segmenting the read counts in the 1 Mb region downstream of the PAS using Bayesian change point analysis from the bcp package in R [82]. Contiguous segmented regions from the PAS with a mean normalized read density greater than 0.01 were combined to give the limits of the potential failed termination region.

### Gene ontology analysis

GO analysis was performed with the goseq package in R [83], which accounts for selection bias in RNA-seq analyses when detecting enrichment of GO classes. Enrichment *P*-values were adjusted using the Benjamini and Hochberg multiple testing correction method [84].

### Data access

RNA-seq data, including tracks suitable for viewing on the UCSC Genome Browser, have been deposited in the ArrayExpress repository [85] under accession E-MTAB-1585.

## Abbreviations

ASO: Antisense oligonucleotide; BRD4: Bromodomain-containing protein 4; ESC: Embryonic stem cell; GFP: Green fluorescent protein; GO: Gene Ontology; GRO-seq: Global run-on sequencing; HEXIM: hexamethylene bis-acetamide inducible 1 mRNA; lincRNA: Long intergenic non-coding RNA; lncRNA: Long non-coding RNA; ncRNA: Non-coding RNA; NSC: Neural stem cell; OPCs: Oligodendrocyte precursor cells; PAS: Polyadenylation site; Pol II: RNA Polymerase II; P-TEFb: Positive transcription elongation factor b; qRT: Quantitative reverse transcription; RNA-seq: RNA sequencing; RPKM: Reads per kilobase per million; SEC: Super elongation complex; SEM: Standard error of the mean; snRNA: Small nuclear RNA; snRNP: Small nuclear ribonucleoprotein complex; TBP: TATA-binding protein; TMM: Trimmed mean of M-values; TSS: Transcription start site; udRNA: Upstream divergent RNA; UTR: Untranslated region.

## Competing interests

The authors declare that they have no competing interest.

## Authors’ contributions

GCB, PA, and TK conceived and designed the experiments in consultation with PE and PB, which were performed by GCB, PA and SCM. GCB, PA, PE, and SR analyzed the data with advice from PB. GCB, PA, PE, SR, SCM, PB and TK contributed reagents, materials, and/or analysis tools. GCB, PA, PE, SR, PB and TK wrote the paper, which was approved by all authors. All authors read and approved the final manuscript.

## Supplementary Material

Additional file 1: Figure S1 **(a)** Quantitative reverse transcription (qRT)-PCR analysis of *7SK* total RNA levels in two independent experiments in which embryonic stem cell (ESCs) were nucleofected with antisense oligonucleotides (ASOs) targeting *7SK* at a position near the 5′ or 3′ end of the RNA (*7SK* 5′ or *7SK* 53′ ASO). Error bars represent standard error of the mean (SEM) for qPCR technical replicates. **(b)** qRT-PCR analysis of *Dll1* total RNA levels when ESCs were nucleofected with *7SK* 5′ and 3′ ASOs. ESCs were replated after nucleofection and collected after 6 hours. Error bars represent SEM for qPCR technical replicates. **(c)** qRT-PCR analysis of *7SK, Dll1*, *Olig2*, and *Hexim1* total RNAs in ESCs after switch to 2iLIF media for several passages. **(d)** qRT-PCR analysis of *Pou5f1* mRNA in ESCs 6 hours after nucleofection with *7SK* 3′ ASO. ESCs were grown in serum (Ser-Ser) or 2iLIF media (2i-2i), or switched from 2iLIF to serum media after nucleofection (2i-Ser). Error bars represent SEM from two independent experiments. **(e)** qRT-PCR analysis of *Pou5f1* nascent RNA in ESCs 6 hours after nucleofection with *7SK* 3′ ASO. Error bars represent SEM from three independent experiments. **(f)** Sample preparation workflow for directional RNA sequencing (RNA-seq). Mouse ESCs were transfected with ASOs, and total RNA was extracted after 6 hours. Two independent experimental sets were used. Total RNA samples were treated with DNAse and depleted for ribosomal RNAs, but not enriched for polyadenylated RNAs. After RNA fragmentation and 5′ and 3′ end polishing, adapters were ligated to the RNAs, in accordance with the instructions of the TruSeq Small RNA sample prep kit (Illumina). The amplified DNA was clustered and run in an Hi-Seq instrument (Illumina) to obtain single-end reads of 50 nucleotides in length. Bioinformatic analysis was performed as described in the Materials and Methods section. **(g)** Breakdown of the number of sequenced reads per sample in the directional RNA-seq, including number of reads mapped to the mouse genome. Click here for file

Additional file 2: Figure S2 **(a)** Ensembl genome browser screenshot showing normalized RNA-seq read coverage (mean of the two biological replicates) at the *Nr4a2* (*Nurr1*) locus. The plus (green) and minus (blue) strand reads are displayed in separate tracks. **(b)** Gene Ontology terms associated with *7SK*-regulated genes. Enrichment *P*-values were adjusted using the Benjamini and Hochberg multiple testing correction method. Click here for file

Additional file 3: Table S1 Genes with altered expression after *7SK* knockdown with two different antisense oligos. Click here for file

Additional file 4: Table S2 All genes with altered expression after *7SK* knockdown. Click here for file

Additional file 5: Figure S3 Box plots and scatter plot depicting log2 fold changes measured by RNA sequencing (RNA-seq) after *7SK* knockdown in mouse ESCs, by counting reads over exons and introns. Of the 438 genes found to be upregulated after *7SK* knockdown, only those with introns are shown (397). Click here for file

Additional file 6: Figure S4 Density scatter plots of normalized read counts for protein-coding genes and surrounding regions. Read counts from experiments in which embryonic stem cell (ESCs) were nucleofected with antisense oligonucleotides (ASOs) targeting the 5′ and 3′ parts of *7SK* (*y*-axis) were plotted versus counts for ESCs nucleofected with scrambled control ASOs (*x*-axis), to illustrate the overall change in expression levels after *7SK* depletion. Color intensity indicates the density of data points. Note the increased read coverage in upstream and downstream regions in *7SK*-depleted samples. Read counts were normalized by the trimmed mean of M-values (TMM) algorithm (see Materials and Methods) and incremented by a pseudocount of 1 to enable visualization on a logarithmic scale. Upstream and downstream 5 kb regions were selected as described in Materials and Methods to avoid inclusion of segments from neighboring genes. Click here for file

Additional file 7: Table S3 Coordinates of genes with failed transcriptional termination regions. Click here for file

Additional file 8: Figure S5 **(a)** Gene-density analysis for failed termination genes. Gene density was computed as the number of unique genes (protein-coding genes and long intergenic non-coding RNAs (lincRNAs) greater than 1 kb long) within a window of +/−100 kb around the end position (final polyadenylation site) of each gene. The resulting distributions are shown for the 1,894 failed transcriptional termination genes (red) versus all other genes (black). In both sets, the majority of genes were found to have 0 to 10 genes within the 200 kb window (failed transcriptional termination genes: mean = 5.949, median = 5; other genes: mean = 5.391, median = 4). **(b)** Box plot depicting log_2_ fold changes by RNA sequencing (RNA-seq) after *7SK* knockdown of downstream sense RNAs and their associated genes in mouse embryonic stem cells (ESCs). **(c)** Gene Ontology terms associated with *7SK*-regulated genes, after background correction. Enrichment *P*-values were adjusted using the Benjamini and Hochberg multiple testing correction method. **(d)** Published poly(A)-negative whole-cell RNA-seq data from human ESCs (ENCODE) showed the presence of upstream divergent RNAs (udRNAs) (purple box). The plus (green) and minus (blue) strand reads are displayed in separate tracks. Click here for file

Additional file 9: Table S4 Genes with altered expression after *7SK* knockdown with antisense oligos and local background adjustment. Click here for file

Additional file 10: Table S5 Upstream divergent RNA (udRNA) transcription units. Click here for file

Additional file 11: Figure S6 **(a)** Venn diagram showing the overlap between upstream divergent RNAs (udRNAs) and antisense transcription start site (TSS)-associated (TSSa) RNAs at the TSS. **(b)** Venn diagram showing that 44.69% (274 of 613) udRNAs overlapping with divergent long non-coding RNAs (lncRNAs) were also upregulated after *7SK* knockdown. **(c)** Venn diagram showing the overlap between genes with failed termination after *7SK* knockdown (‘hotspot’ genes) and *7SK*-regulated udRNAs. **(d)** Quantitative reverse transcription (qRT)-PCR analysis of *Hexim1* total RNA, and *Dll1* nascent RNA, 6 hours after nucleofection of embryonic stem cell (ESCs) with scrambled *7SK* 3′ antisense oligonucleotides (ASOs) targeting the 3′ segments of *7SK*, in the presence or absence of I-BET151. Error bars represent standard error of the mean (SEM) from two to three independent experiments. **(e)** Box plot depicting log_2_ fold changes measured by RNA sequencing (RNA-seq) after *7SK* knockdown of udRNAs and their associated genes in mouse ESCs, using either *7SK* 5′ or *7SK* 3′ ASO data for udRNA detection. Click here for file

Additional file 12: Table S6 Gene Ontology analysis of upstream divergent RNAs (udRNAs). Click here for file

Additional file 13: Figure S7 **(A)** Quantitative reverse transcription (qRT)-PCR analysis of *7SK* and *Olig2* total RNA, and *Sox9* mRNA levels after nucleofection of neural stem cells (NSCs) with *7SK* 5′ and 3′ antisense oligonucleotides (ASOs), compared with scrambled and green fluorescent protein (GFP) ASOs (control; CTRL). NSCs were replated after nucleofection, and collected after 6 and 24 hours. Error bars represent standard error of the mean (SEM) for two independent experiments. **(B, C)** qRT-PCR analysis of **(B)***Hes1, Irx2*, and *Nr2a4* nascent RNA and **(C)***Hes1* and *Rbm34* udRNA after nucleofection of NSCs with *7SK* 3′ ASOs compared with scrambled ASO (CTRL). NSCs were replated after nucleofection, and collected after 6 hours. Error bars represent standard deviation (SD) of qPCR technical replicates. **(D, E)** qRT-PCR analysis of **(D)***7SK, Sox2, Hexim1*, and *Olig2* total RNA, and *Dll1*, *CNP*, and *MBP* nascent RNA and **(E)***Rbm34, hnRNPL*, and *Hes1* udRNA, *Sox8OT* (AK079380) total RNA, and *Sox10OT* (Gm10863) spliced RNA after lipofection of Oli-neu oligodendrocyte precursor cells (OPCs) with *7SK* 3′ ASOs compared with scrambled ASOs (CTRL). OPCs were collected after 6 and 24 hours. Error bars represent SEM for three independent experiments. Click here for file

Additional file 14: Table S7 Sequence of quantitative reverse transcription (qRT)-PCR primers and antisense oligonucleotides. Click here for file
