# Supplementary material for: The non-coding snRNA 7SK controls transcriptional termination, poising, and bidirectionality in embryonic stem cells
Source: Genome Biol. 2013 Sep 17;14(9):R98. doi: 10.1186/gb-2013-14-9-r98 (PMC4053805; doi:10.1186/gb-2013-14-9-r98)
Supplement: Additional file 1: Figure S1 — (a) Quantitative reverse transcription (qRT)-PCR analysis of 7SK total RNA levels in two independent experiments in which embryonic stem cell (ESCs) were nucleofected with antisense oligonucleotides (ASOs) targeting 7SK at a position near the 5′ or 3′ end of the RNA (7SK 5′ or 7SK 53′ ASO). Error bars represent standard error of the mean (SEM) for qPCR technical replicates. (b) qRT-PCR analysis of Dll1 total RNA levels when ESCs were nucleofected with 7SK 5′ and 3′ ASOs. ESCs were replated after nucleofection and collected after 6 hours. Error bars represent SEM for qPCR technical replicates. (c) qRT-PCR analysis of 7SK, Dll1, Olig2, and Hexim1 total RNAs in ESCs after switch to 2iLIF media for several passages. (d) qRT-PCR analysis of Pou5f1 mRNA in ESCs 6 hours after nucleofection with 7SK 3′ ASO. ESCs were grown in serum (Ser-Ser) or 2iLIF media (2i-2i), or switched from 2iLIF to serum media after nucleofection (2i-Ser). Error bars represent SEM from two independent experiments. (e) qRT-PCR analysis of Pou5f1 nascent RNA in ESCs 6 hours after nucleofection with 7SK 3′ ASO. Error bars represent SEM from three independent experiments. (f) Sample preparation workflow for directional RNA sequencing (RNA-seq). Mouse ESCs were transfected with ASOs, and total RNA was extracted after 6 hours. Two independent experimental sets were used. Total RNA samples were treated with DNAse and depleted for ribosomal RNAs, but not enriched for polyadenylated RNAs. After RNA fragmentation and 5′ and 3′ end polishing, adapters were ligated to the RNAs, in accordance with the instructions of the TruSeq Small RNA sample prep kit (Illumina). The amplified DNA was clustered and run in an Hi-Seq instrument (Illumina) to obtain single-end reads of 50 nucleotides in length. Bioinformatic analysis was performed as described in the Materials and Methods section. (g) Breakdown of the number of sequenced reads per sample in the directional RNA-seq, including number of reads mapped to the mouse g [file gb-2013-14-9-r98-S1.pdf]

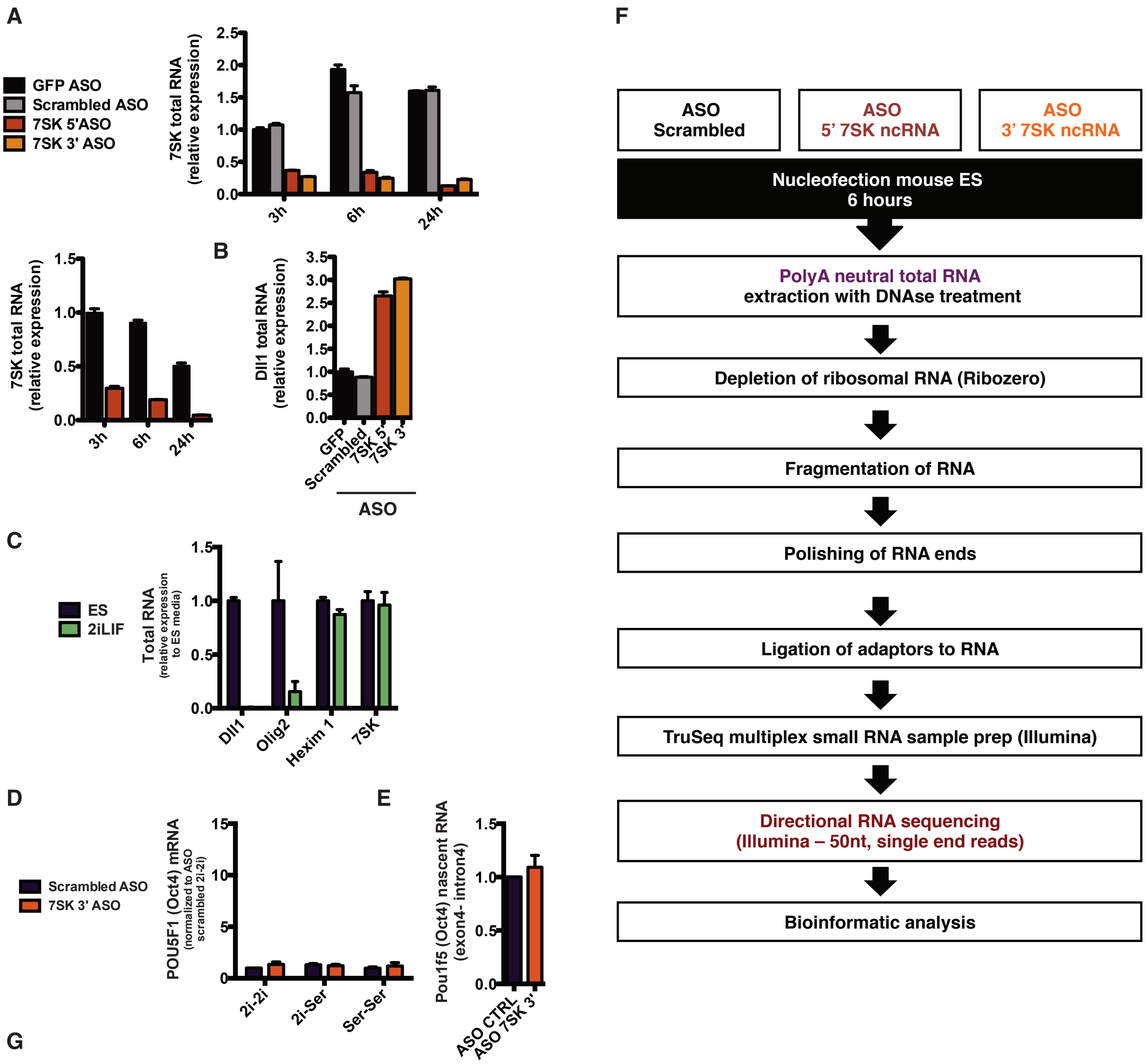

G

Reads	Ctrl experiment 1	ASO 7SK 5' experiment 1	ASO 7SK 3' experiment 1	Ctrl experiment 2	ASO 7SK 5' experiment 2	ASO 7SK 3' experiment 2
Uniquely mapped	13,827,079	10,355,781	5,356,746	4,810,847	6,202,222	13,383,204
Ambiguously mapped	8,919,853	5,885,881	2,984,129	3,641,993	4,171,779	8,122,804
Ribosomal RNA	10,579,416	8,805,427	3,681,418	3,654,151	3,728,745	7,816,072
Unmapped	31,477,558	29,316,123	10,304,750	9,784,614	10,261,041	22,720,518
Short (<20 nt)	15,460,393	13,143,006	5,210,801	4,784,537	5,058,887	13,329,356
Total	80,264,299	67,506,218	27,537,844	26,676,142	29,422,674	65,371,954

Supplementary Figure 1

(a) qRT-PCR analysis of *7SK* total RNA levels in two independent experiments where ES cells were nucleofected with ASOs targeting *7SK* at a position near the 5' or 3' end of the RNA (7SK 5' or 3' ASO). Error bars represent SEM for qPCR technical replicates. **(b)** qRT-PCR analysis of *Dll1* total RNA levels when ES cells were nucleofected with 7SK 5' and 3' ASOs. ES cells were replated after nucleofection and collected after 6h. Error bars represent SEM for qPCR technical replicates. **(c)** qRT-PCR analysis of *7SK,Dll1*, *Olig2* and *Hexim1 total RNAs* in ES cells upon switch to 2iLIF media for several passages.
(d) qRT-PCR analysis of *Pou5f1* mRNA in ES cells 6h after nucleofection with 7SK 3' ASO. ES cell were expanded in serum (Ser-Ser), 2iLIF media (2i-2i), or switched from 2iLIF to serum media after nucleofection (2i-Ser). Error bars represent SEM from 2 independent experiments. **(e)** qRT-PCR analysis of *Pou5f1* nascent RNA in ES cells 6h after nucleofection with 7SK 3' ASO. Error bars represent SEM from 3 independent experiments. **(f)** Sample preparation workflow for directional RNA-Sequencing. Mouse ES cells were transfected with ASOs and total RNA was extracted after 6 hours. Two independent experimental sets were used. Total RNA samples were treated with DNAse and depleted for ribossomal RNAs, but not enriched for poly-adenylated RNAs. Upon RNA fragmentation and 5' and 3' end polishing, adapters were ligated to the RNAs, according to the TruSeq Small RNA sample prep kit (Illumina).The amplified DNA was clustered and run in an Hi-Seq instrument to obtain single-end reads of 50 nts in length. Bioinformatic analysis was performed as described in the Material and Method. **(g)** Break-down of number of sequenced reads per sample in the directional RNA-Sequencing, including number of reads mapped to the mouse genome.
