# Supplementary material for: The non-coding snRNA 7SK controls transcriptional termination, poising, and bidirectionality in embryonic stem cells
Source: Genome Biol. 2013 Sep 17;14(9):R98. doi: 10.1186/gb-2013-14-9-r98 (PMC4053805; doi:10.1186/gb-2013-14-9-r98)
Supplement: Additional file 2: Figure S2 — (a) Ensembl genome browser screenshot showing normalized RNA-seq read coverage (mean of the two biological replicates) at the Nr4a2 (Nurr1) locus. The plus (green) and minus (blue) strand reads are displayed in separate tracks. (b) Gene Ontology terms associated with 7SK-regulated genes. Enrichment P-values were adjusted using the Benjamini and Hochberg multiple testing correction method. [file gb-2013-14-9-r98-S2.pdf]

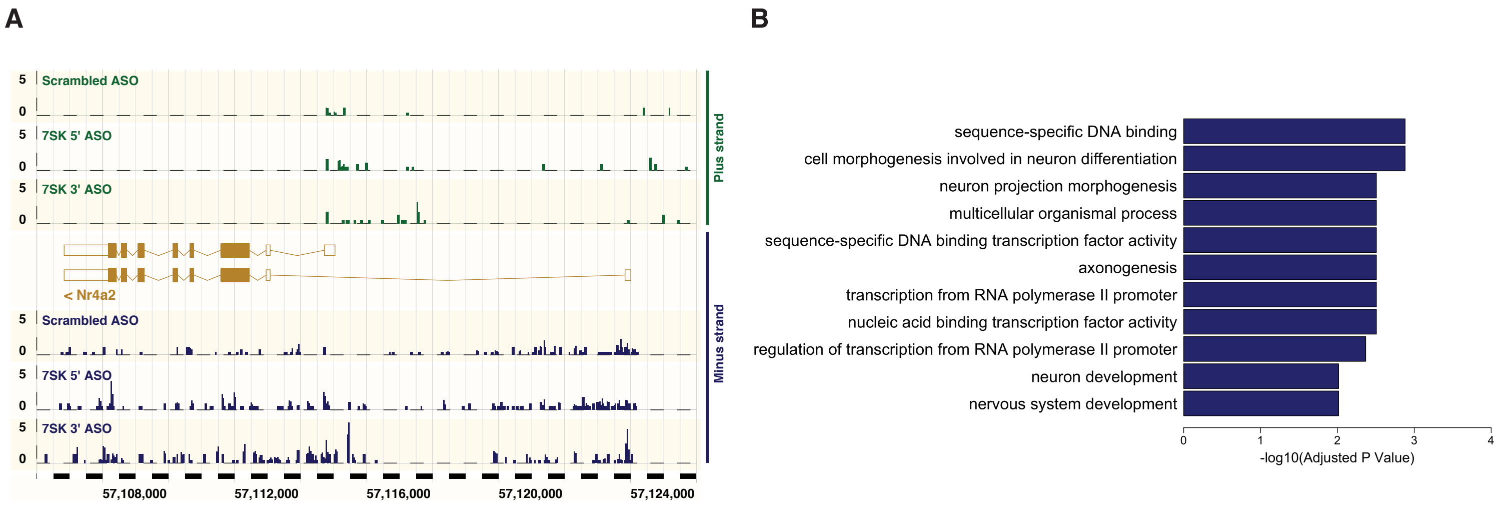

Supplementary Figure 2

(a) Ensembl genome browser screenshot showing normalized RNA-seq read coverage (mean of the two biological replicates) at the *Nr4a2* (*Nurr1*) locus. Plus (green) and minus (blue) strand reads are displayed in separate tracks.
(b) Gene ontology terms associated with 7SK-regulated genes. Enrichment *P*-values were adjusted using the Benjamini and Hochberg multiple testing correction method.
