# Supplementary material for: The non-coding snRNA 7SK controls transcriptional termination, poising, and bidirectionality in embryonic stem cells
Source: Genome Biol. 2013 Sep 17;14(9):R98. doi: 10.1186/gb-2013-14-9-r98 (PMC4053805; doi:10.1186/gb-2013-14-9-r98)
Supplement: Additional file 5: Figure S3 — Box plots and scatter plot depicting log2 fold changes measured by RNA sequencing (RNA-seq) after 7SK knockdown in mouse ESCs, by counting reads over exons and introns. Of the 438 genes found to be upregulated after 7SK knockdown, only those with introns are shown (397). [file gb-2013-14-9-r98-S5.pdf]

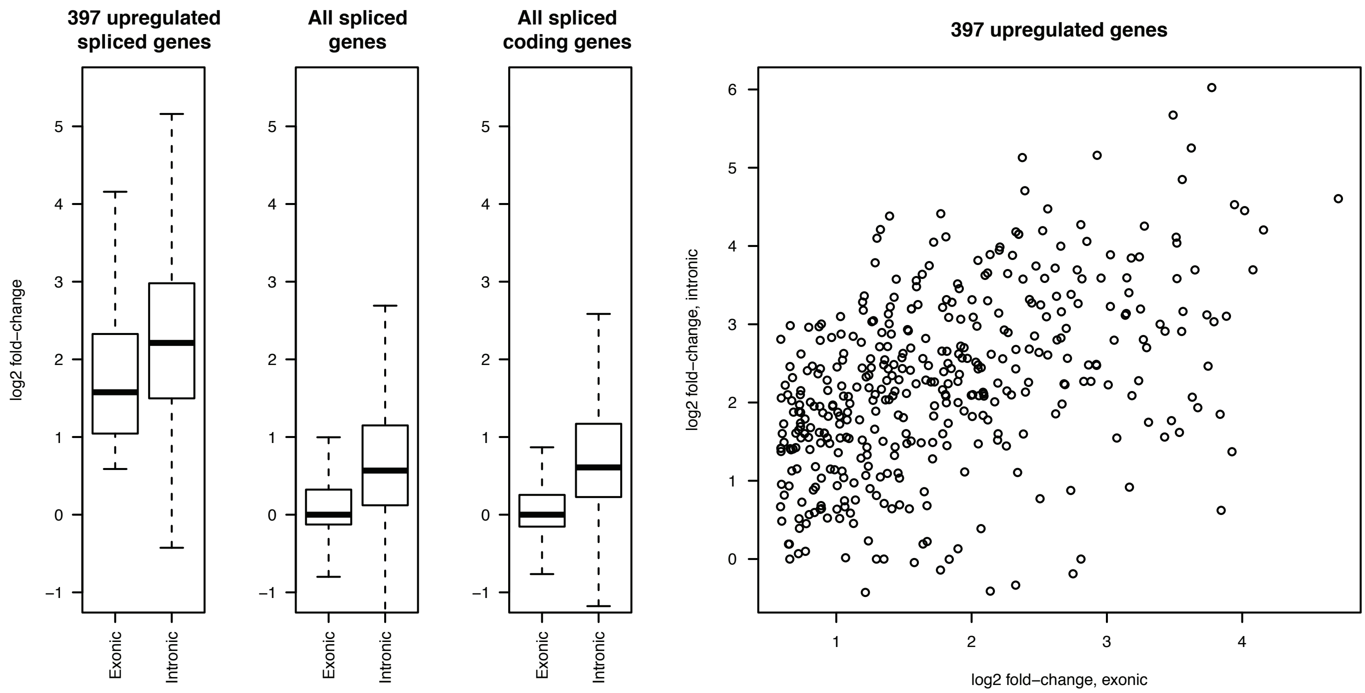

Supplementary Figure 3
Box plots and scatter plot depicting log_2 fold changes measured by RNA-seq upon *7SK* knockdown in mouse ES cells, by counting reads over exons and introns. Of the 438 genes found to be upregulated upon 7SK knockdown, only those with introns are shown (397).
