# Supplementary material for: The non-coding snRNA 7SK controls transcriptional termination, poising, and bidirectionality in embryonic stem cells
Source: Genome Biol. 2013 Sep 17;14(9):R98. doi: 10.1186/gb-2013-14-9-r98 (PMC4053805; doi:10.1186/gb-2013-14-9-r98)
Supplement: Additional file 6: Figure S4 — Density scatter plots of normalized read counts for protein-coding genes and surrounding regions. Read counts from experiments in which embryonic stem cell (ESCs) were nucleofected with antisense oligonucleotides (ASOs) targeting the 5′ and 3′ parts of 7SK (y-axis) were plotted versus counts for ESCs nucleofected with scrambled control ASOs (x-axis), to illustrate the overall change in expression levels after 7SK depletion. Color intensity indicates the density of data points. Note the increased read coverage in upstream and downstream regions in 7SK-depleted samples. Read counts were normalized by the trimmed mean of M-values (TMM) algorithm (see Materials and Methods) and incremented by a pseudocount of 1 to enable visualization on a logarithmic scale. Upstream and downstream 5 kb regions were selected as described in Materials and Methods to avoid inclusion of segments from neighboring genes. [file gb-2013-14-9-r98-S6.pdf]

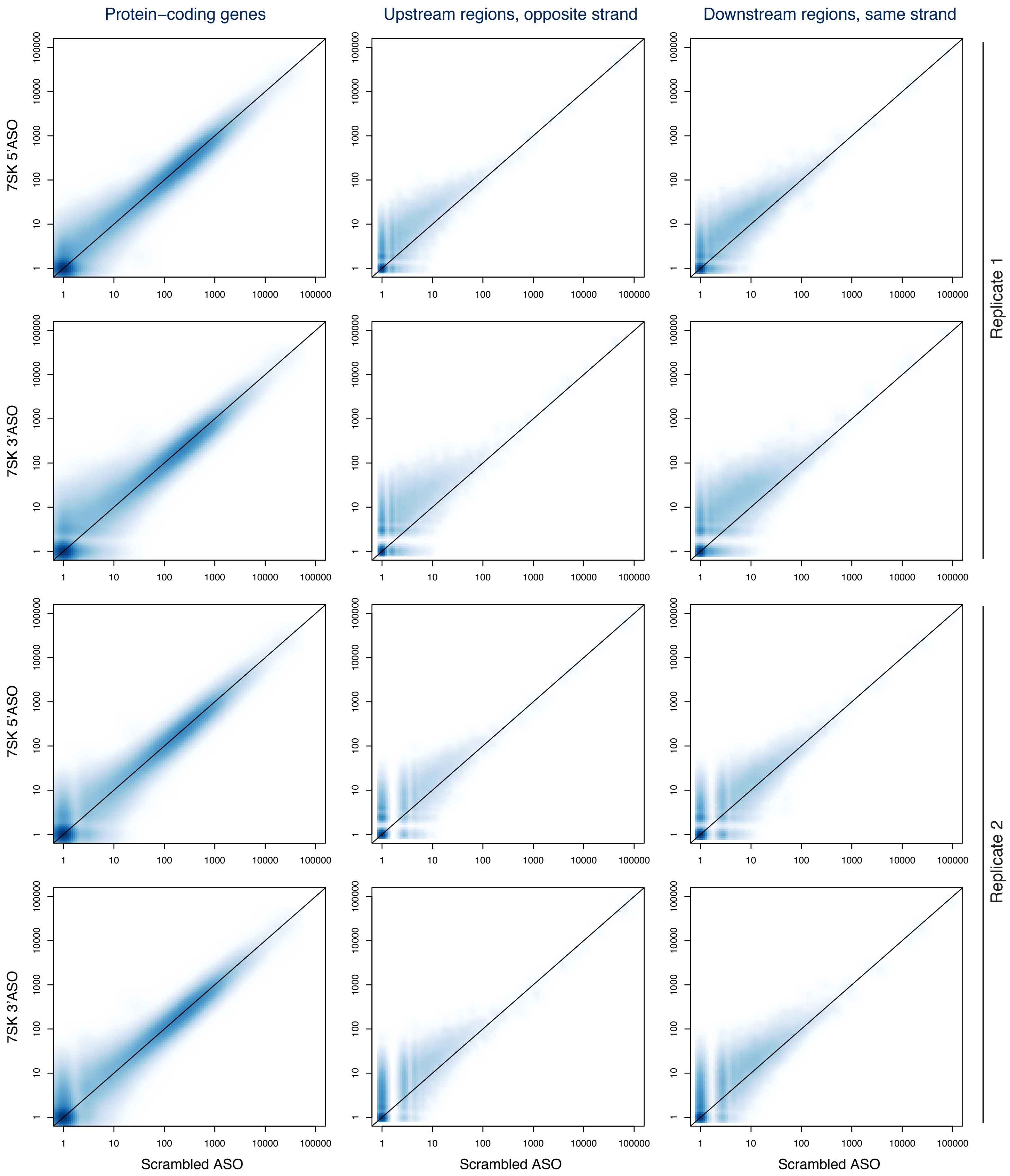

Supplementary Figure 4
Density scatter plots of normalized read counts for protein-coding genes and surrounding regions. Read counts from experiments where ES cells were nucleofected with ASOs targeting the 5' and 3' parts of 7SK (y axis) are plotted versus counts for ES cells nucleofected with scrambled control ASOs (x axis), to illustrate the overall change in expression levels following 7SK depletion. Color intensity indicates the density of data points. Note the elevated read coverage in up- and downstream regions in 7SK-depleted samples. Read counts were normalized by the TMM algorithm (see Material and Methods) and incremented by a pseudocount of one to enable visualization on a logarithmic scale. Up- and downstream 5 kb regions were selected as described in Material and Methods to avoid inclusion of segments from neighboring genes.
