# Supplementary material for: The non-coding snRNA 7SK controls transcriptional termination, poising, and bidirectionality in embryonic stem cells
Source: Genome Biol. 2013 Sep 17;14(9):R98. doi: 10.1186/gb-2013-14-9-r98 (PMC4053805; doi:10.1186/gb-2013-14-9-r98)
Supplement: Additional file 8: Figure S5 — (a) Gene-density analysis for failed termination genes. Gene density was computed as the number of unique genes (protein-coding genes and long intergenic non-coding RNAs (lincRNAs) greater than 1 kb long) within a window of +/−100 kb around the end position (final polyadenylation site) of each gene. The resulting distributions are shown for the 1,894 failed transcriptional termination genes (red) versus all other genes (black). In both sets, the majority of genes were found to have 0 to 10 genes within the 200 kb window (failed transcriptional termination genes: mean = 5.949, median = 5; other genes: mean = 5.391, median = 4). (b) Box plot depicting log2 fold changes by RNA sequencing (RNA-seq) after 7SK knockdown of downstream sense RNAs and their associated genes in mouse embryonic stem cells (ESCs). (c) Gene Ontology terms associated with 7SK-regulated genes, after background correction. Enrichment P-values were adjusted using the Benjamini and Hochberg multiple testing correction method. (d) Published poly(A)-negative whole-cell RNA-seq data from human ESCs (ENCODE) showed the presence of upstream divergent RNAs (udRNAs) (purple box). The plus (green) and minus (blue) strand reads are displayed in separate tracks. [file gb-2013-14-9-r98-S8.pdf]

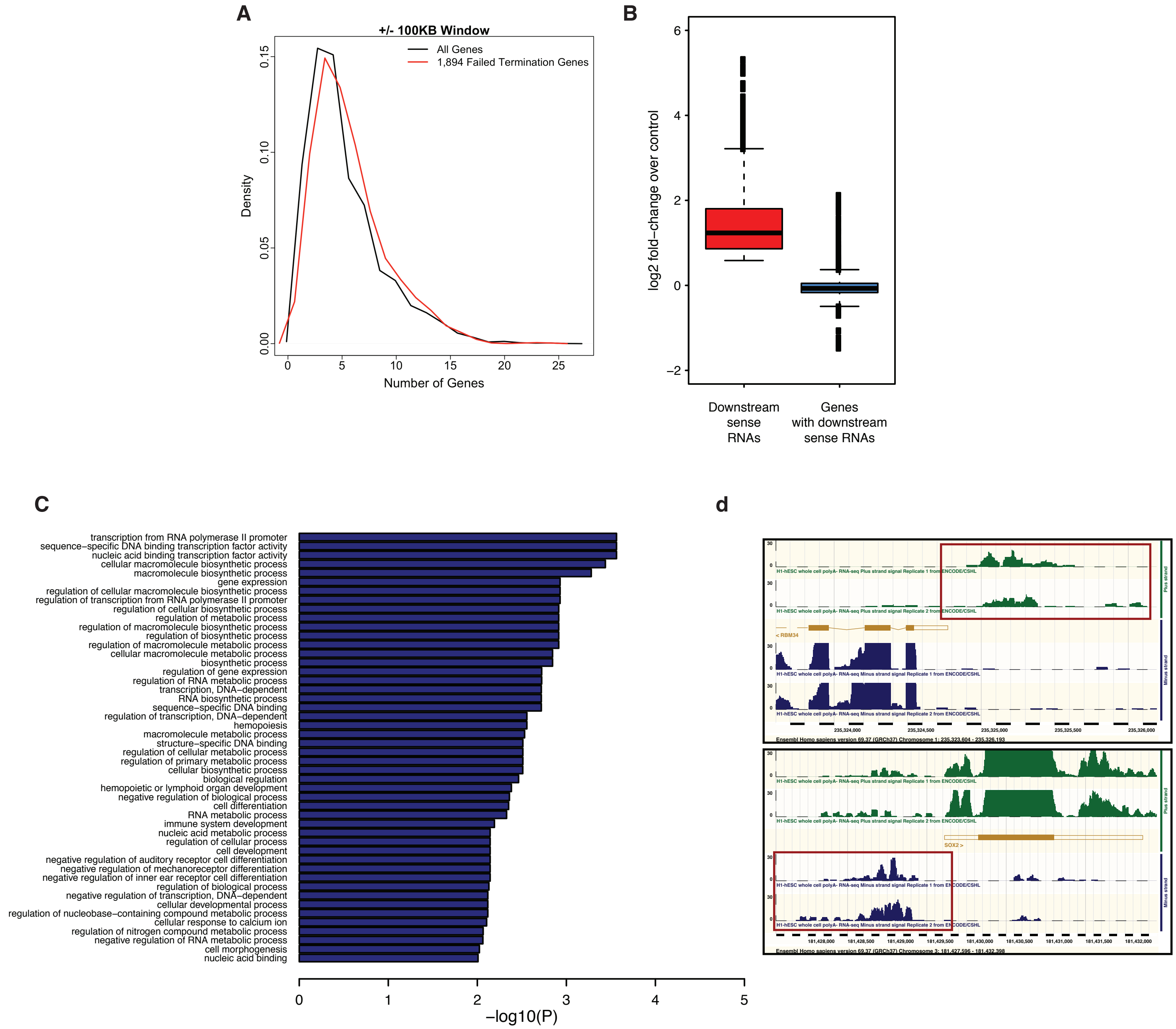

Supplementary Figure 5

(a) Gene density analysis for failed termination genes. Gene density was computed as the number of unique genes (protein -coding genes and lincRNAs greater than 1 kb) within a window of +/-100 kb around the end position (last polyadenylation site) of each gene. Resulting distributions are shown for the 1,894 failed transcriptional termination genes (red) vs all other genes (black). In both sets, the majority of genes have 0-10 genes within the 200 kb window (failed transcriptional termination genes: mean = 5.949, median = 5; other genes: mean = 5.391, median = 4). (**b**) Box plot depicting log2 fold changes by RNA-seq upon 7SK knockdown in mouse ES cells of downstream sense RNAs and their associated genes. **(c)** Gene ontology terms associated with 7SK-regulated genes, after background correction. Enrichment *P*-values were adjusted using the Benjamini and Hochberg multiple testing correction method. **(d)** Published poly(A)-negative whole cell RNA-seq data from human ES cells (ENCODE) show presence of udRNAs (purple box). Plus (green) and minus (blue) strand reads are displayed in separate tracks.
