# Supplementary material for: The non-coding snRNA 7SK controls transcriptional termination, poising, and bidirectionality in embryonic stem cells
Source: Genome Biol. 2013 Sep 17;14(9):R98. doi: 10.1186/gb-2013-14-9-r98 (PMC4053805; doi:10.1186/gb-2013-14-9-r98)
Supplement: Additional file 11: Figure S6 — (a) Venn diagram showing the overlap between upstream divergent RNAs (udRNAs) and antisense transcription start site (TSS)-associated (TSSa) RNAs at the TSS. (b) Venn diagram showing that 44.69% (274 of 613) udRNAs overlapping with divergent long non-coding RNAs (lncRNAs) were also upregulated after 7SK knockdown. (c) Venn diagram showing the overlap between genes with failed termination after 7SK knockdown (‘hotspot’ genes) and 7SK-regulated udRNAs. (d) Quantitative reverse transcription (qRT)-PCR analysis of Hexim1 total RNA, and Dll1 nascent RNA, 6 hours after nucleofection of embryonic stem cell (ESCs) with scrambled 7SK 3′ antisense oligonucleotides (ASOs) targeting the 3′ segments of 7SK, in the presence or absence of I-BET151. Error bars represent standard error of the mean (SEM) from two to three independent experiments. (e) Box plot depicting log2 fold changes measured by RNA sequencing (RNA-seq) after 7SK knockdown of udRNAs and their associated genes in mouse ESCs, using either 7SK 5′ or 7SK 3′ ASO data for udRNA detection. [file gb-2013-14-9-r98-S11.pdf]

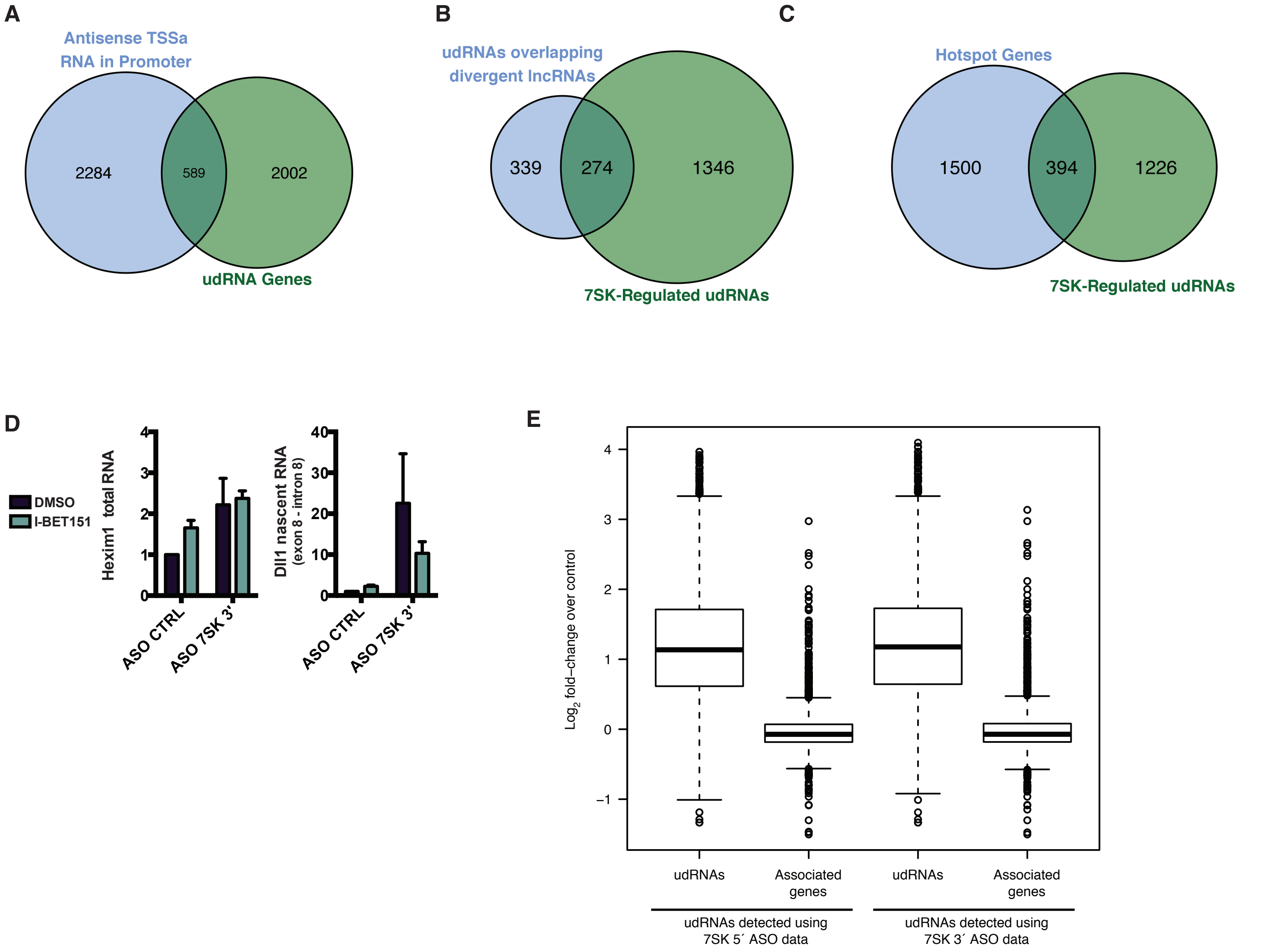

Supplementary Figure 6 (a) Venn diagram showing the overlap between udRNAs and antisense TSS-associated (TSSa) RNAs at the TSS. **(b)** Venn diagram showing that 44.69% (274 out of 613) of udRNAs overlapping with divergent lncRNAs were also upregulated upon 7SK knockdown. **(c)** Venn diagram showing the overlap between genes with failed termination after 7SK knockdown ("hotspot" genes) and 7SK regulated udRNAs.(**d**) qRT-PCR analysis of *Hexim1* total RNA, and *Dll1* nascent RNA, 6h after nucleofection of ES cells with scrambled of 7SK 3´ ASOs targeting the 3´ segments of 7SK, in the presence or absence of I-BET151. Error bars represent standard error of the mean (SEM) from 2-3 independent experiments. (**e**) Box plot depicting log2 fold changes measured by RNA-seq upon 7SK knockdown in mouse ES cells of udRNAs and their associated genes, using either 7SK 5´and 3´ASO data for udRNA detection.
