# Supplementary material for: The non-coding snRNA 7SK controls transcriptional termination, poising, and bidirectionality in embryonic stem cells
Source: Genome Biol. 2013 Sep 17;14(9):R98. doi: 10.1186/gb-2013-14-9-r98 (PMC4053805; doi:10.1186/gb-2013-14-9-r98)
Supplement: Additional file 13: Figure S7 — (A) Quantitative reverse transcription (qRT)-PCR analysis of 7SK and Olig2 total RNA, and Sox9 mRNA levels after nucleofection of neural stem cells (NSCs) with 7SK 5′ and 3′ antisense oligonucleotides (ASOs), compared with scrambled and green fluorescent protein (GFP) ASOs (control; CTRL). NSCs were replated after nucleofection, and collected after 6 and 24 hours. Error bars represent standard error of the mean (SEM) for two independent experiments. (B, C) qRT-PCR analysis of (B)Hes1, Irx2, and Nr2a4 nascent RNA and (C)Hes1 and Rbm34 udRNA after nucleofection of NSCs with 7SK 3′ ASOs compared with scrambled ASO (CTRL). NSCs were replated after nucleofection, and collected after 6 hours. Error bars represent standard deviation (SD) of qPCR technical replicates. (D, E) qRT-PCR analysis of (D)7SK, Sox2, Hexim1, and Olig2 total RNA, and Dll1, CNP, and MBP nascent RNA and (E)Rbm34, hnRNPL, and Hes1 udRNA, Sox8OT (AK079380) total RNA, and Sox10OT (Gm10863) spliced RNA after lipofection of Oli-neu oligodendrocyte precursor cells (OPCs) with 7SK 3′ ASOs compared with scrambled ASOs (CTRL). OPCs were collected after 6 and 24 hours. Error bars represent SEM for three independent experiments. [file gb-2013-14-9-r98-S13.pdf]

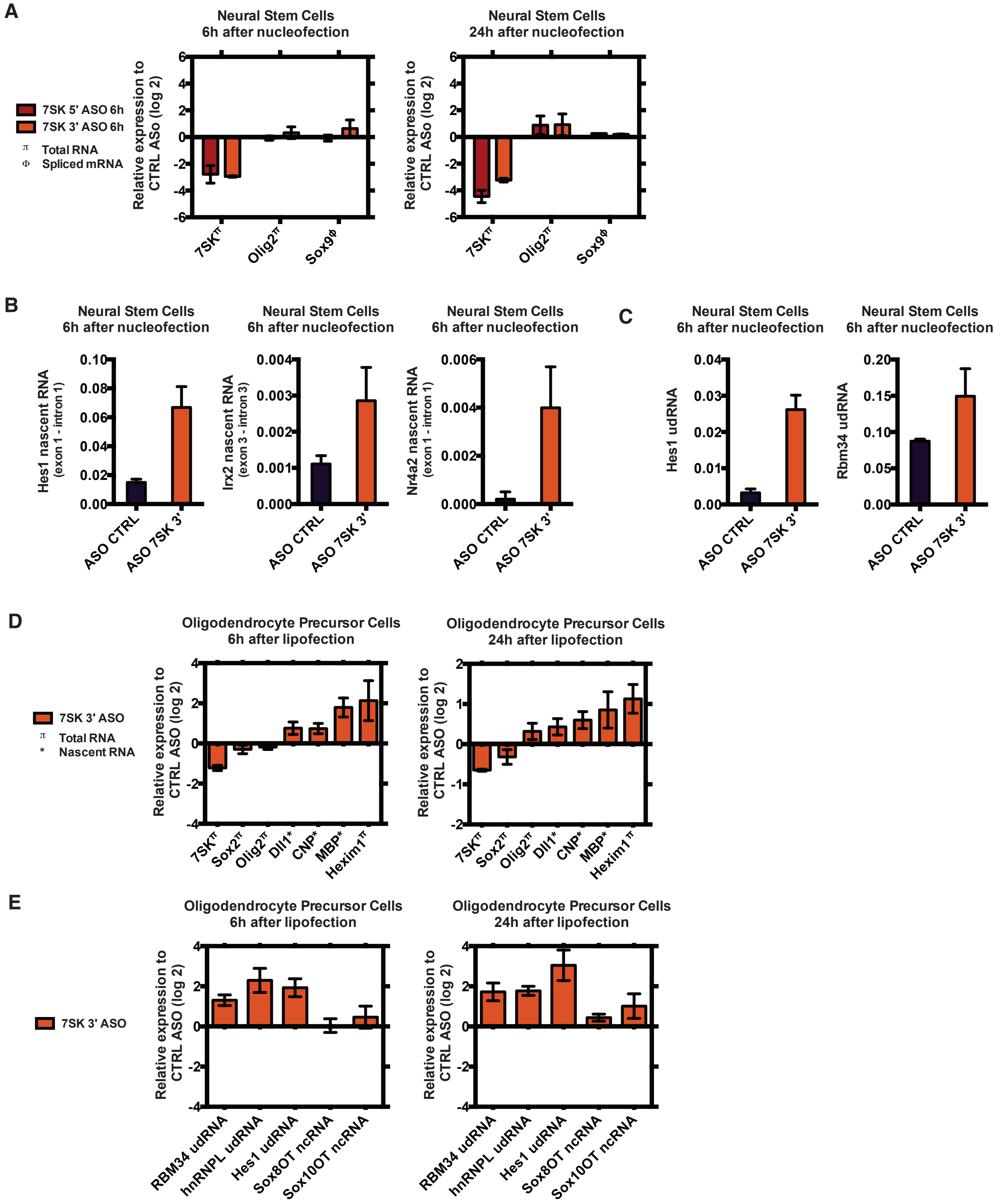

Supplementary Figure 7

(a) qRT-PCR analysis of *7SK* and *Olig2* total RNA, and *Sox9* mRNA levels upon nucleofection of NS cells with 7SK 5' and 3' ASOs compared to scrambled and GFP ASOs (CTRL). NS cells were replated after nucleofection and collected after 6 and 24hours. Error bars represent SEM for 2 independent experiments. **(b,c)** qRT-PCR analysis of *Hes1, Irx2* and *Nr2a4* nascent RNA (b) and *Hes1* and *Rbm34* udRNA (c) upon nucleofection of NS cells with 7SK 3' ASOs compared to scrambled ASO (CTRL). NS cells were replated after nucleofection and collected after 6 hours. Error bars represent SD of qPCR technical replicates. **(d, e)** qRT-PCR analysis of *7SK, Sox2, Hexim1* and *Olig2* total RNA, and *Dll1*, *CNP* and *MBP* nascent RNA (d) and *Rbm34, hnRNPL* and *Hes1* udRNA, *Sox8OT (*AK079380*)* total and *Sox10OT* (Gm10863) spliced RNA (e) upon lipofection of Oli-neu OPC cells with *7SK* 3' ASOs compared to scrambled ASO (CTRL). OPCs were collected after 6 and 24 hours. Error bars represent SEM for 3 independent experiments.
